# Metabolic enzyme PFKFB3 mediates matrix stiffness‐potentiated tumour growth and radiotherapeutic resistance in HCC

**DOI:** 10.1002/ctm2.70509

**Published:** 2025-11-25

**Authors:** Mimi Wang, Jiajun Li, Jiali Qian, Xi Zhang, Miao Li, Yingying Zhao, Zhiming Wang, Kun Guo, Dongmei Gao, Yan Zhao, Rongxin Chen, Zhenggang Ren, Taiwei Sun, Fan Wang, Jiefeng Cui

**Affiliations:** ^1^ Liver Cancer Institute Zhongshan Hospital, Fudan University & Key Laboratory of Carcinogenesis and Cancer Invasion Ministry of Education Shanghai PR China; ^2^ Department of Endocrinology Huashan Hospital Fudan University Shanghai PR China; ^3^ Department of Oncology Zhongshan Hospital Fudan University Shanghai PR China; ^4^ Department of Radiotherapy Zhongshan Hospital Fudan University Shanghai PR China; ^5^ Department of Oncology, Cancer Center Shanghai General Hospital, Shanghai Jiao Tong University School of Medicine Shanghai PR China

**Keywords:** DNA repair, hepatocellular carcinoma, matrix stiffness, PFKFB3

## Abstract

**Background:**

Although the contribution of matrix stiffness to aggravating the malignant features of HCC cells has been well documented, the effects of matrix stiffness on chemoradiotherapy resistance and its underlying mechanism remain largely elusive.

**Methods:**

To delineate the role of matrix stiffness in HCC progression, we engineered novel in vivo animal models with defined liver stiffness and a complementary tunable hydrogel culture system. This integrated approach enabled a comprehensive investigation into how biomechanical cues modulate HCC cell proliferation and DNA repair both in vitro and in vivo.

**Results:**

High stiffness stimulation noticeably enhanced cell proliferation and cell survival from DNA damage through changing the expression and distribution of metabolic enzyme PFKFB3. Specifically, high stiffness stimulation prominently suppressed PFKFB3 ubiquitination by downregulating E3 ubiquitin ligase NEDD4, and then increased the stability of PFKFB3 protein to enhance glycolysis, ultimately promoted HCC growth. Meanwhile, high matrix stiffness stimulation also effectively strengthened the DNA damage repair ability of irradiated HCC cells, and PFKFB3 nuclear translocation mediated in matrix stiffness‐regulated DNA damage repair by interacting with Ku70.

**Conclusions:**

Our results delineate a PFKFB3‐mediated pathway that underpins how increased matrix stiffness potentiates HCC growth and compromises radiotherapy efficacy. These findings not only highlight the contribution of matrix stiffness to tumor growth and DNA damage repair in HCC, but also disclose a previously unidentified nonmetabolic function of PFKFB3.

**Key points:**

- Increased matrix stiffness significantly promoted glycolysis in HCC cells via upregulating PFKFB3 expression.
- High stiffness stimulation suppressed PFKFB3 ubiquitination by downregulating E3 ubiquitin ligase NEDD4 expression.
- PFKFB3 participated in DNA damage repair by translocating into nuclear and interacting with Ku70, which strengthened by matrix stiffness.

## Graphical abstract

This study proposes a stiffness‐triggered pathway that co‐opts PFKFB3 expression and localization to fuel HCC growth and compromise radiotherapy efficacy. Specifically, matrix stiffness suppresses PFKFB3 ubiquitination by downregulating E3 ubiquitin ligase NEDD4 expression, and then enhances the stablity of PFKFB3 protein to increase glycolysis. Simultaneously, matrix stiffness strengthens the DNA damage repair ability of irradiated HCC cells through PFKFB3 nuclear translocation and interacting with Ku70.

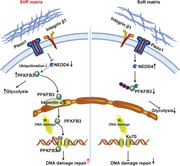

## 1 INTRODUCTION

Chemoradiotherapy resistance has become a major clinical challenge in the treatment of advanced solid tumour.^1^, ^2^ DNA damage is a common mechanism of radiotherapy and cytotoxic chemotherapy in killing tumour cells, and the ability to repair DNA damage often determines the efficacy and sensitivity of tumour radiotherapy and cytotoxic chemotherapy.^3^, ^4^ So, exploring new approach to weaken the ability of DNA damage repair is undoubtedly of great significance for conquering this clinical challenge. In addition to the intrinsic factors of tumour cells themselves, such as gene mutation, metabolic reprogramming, oxidative stress, abnormal cell cycle and apoptosis, DNA damage repair ability and tumour stem cells,^5^, ^6^, ^7^, ^8^ the external factors in microenvironment including physicochemical stimuli and non‐tumour cells are also involved in modulating tumour cell sensitivity to radiotherapy and chemotherapy.^9^, ^10^, ^11^ Hypoxia upregulates the expression of vascular endothelial growth factor, and thereby augments tumour resistance to radiation damage and tumour survival.^12^ Similarly, the accumulation of lactic acid exerts a positive impact on the resistance to tumour radiotherapy and chemotherapy.^13^, ^14^ Besides, enhanced immunosuppressive microenvironment caused by the infiltrated myeloid‐derived suppressor cells also boosts tumour resistance to radiotherapy.^15^, ^16^ These findings strongly address the role of biochemical signals in regulating DNA damage repair and altering the efficacy of tumour radiotherapy and cytotoxic chemotherapy. However, the contribution of biomechanical signals in tumour microenvironment to chemoradiotherapy resistance and DNA damage repair remained largely unexplored.

Substantial evidence has validated that matrix stiffness, a prominent biomechanical feature of solid tumour, can prominently aggravate the malignant features of tumour cells (proliferation, migration, invasion and metastasis, etc.) and facilitate tumour progression.^17^, ^18^, ^19^, ^20^ On the other hand, matrix stiffness may influence or regulate chemoradiotherapy resistance. A small amount of research demonstrates that matrix stiffness enhances the resistance of ovarian cancer cells to platinum‐induced DNA damage,^21^ and CNN1^hi^CAFs‐mediated matrix stiffening contributes to 5‐Fu resistance in gastric cancer.^22^ Similarly, matrix stiffness seems to impair the sensitivity of cytotoxic chemotherapy in HCC cells.^23^ Although nascent evidence points to associations between tissue rigidity and chemoresistance, the direct mechanistic linkage through which matrix stiffness governs DNA damage repair efficiency awaits definitive characterization.

Sustaining proliferation is one of the hallmarks of tumour cells. Aerobic glycolysis as the main energy provider meets the energy needs of the rapid tumour proliferation.^24^ The expression level and activity of glycolytic enzymes usually reflect the state of glycolysis in tumour cells.^25^, ^26^ Additionally, different from their classic enzyme function, metabolic enzymes have also non metabolic functions to control cellular activities, cell survival and diseases progression through nuclear translocation.^27^ Considering the potential effects of matrix stiffness on chemoradiotherapy resistance, and the function of glycolytic enzymes on cell proliferation and survival, we speculated that matrix stiffness may alter the expression and distribution of glycolytic enzymes, and thereby promote tumour growth and radiotherapeutic resistance in HCC.

In the present study, we established novel animal models with controlled liver stiffness and a complementary tunable hydrogel culture system to explore how matrix stiffness modulated HCC cell proliferation and DNA repair. Our results demonstrated that matrix stiffness significantly promoted DNA repair and conferred radiotherapeutic resistance in HCC. Furthermore, we identified a noncanonical, non‐metabolic role for the glycolytic enzyme PFKFB3 in mediating this mechanosensitive pathway.

## 2 MATERIALS AND METHODS

### 2.1 Cell culture and HCC tissue samples

MHCC97H cells were obtained from Liver Cancer Institute of Zhongshan Hospital, Fudan University in Shanghai. Hep3B cells and McA‐RH7777 cells were purchased from Cell Bank of Shanghai Institute of Biochemistry and Cell Biology and Shanghai Zishi Biotech Co., Ltd., respectively. MHCC97H cells and McA‐RH7777 cells were cultured in Dulbecco's Modified Eagle's Medium (DMEM, Gibco) with 10% foetal bovine serum (FBS, Biowest) and 1% penicillin/streptomycin (Gibco), while Hep3B cells were cultured in Minimum Essential Medium (Gibco) with 12.5% FBS and 1% penicillin/streptomycin.

Clinical data and HCC tissue samples of 87 HCC patients, who underwent curative surgical resection at Department of Liver Surgery, Zhongshan Hospital of Fudan University in 2008, were collected and obtained. The patients were followed up until December 2022. Table 1 displays the clinicopathologic features of the patients and the statistical analysis included in the study. HCC tissues and clinical data used in the study were approved by the Ethics Committee of the Zhongshan Hospital of Fudan University (B2024‐359R).

**TABLE 1:** Relationship between PFKFB3 expression and clinicopathological features in tumour tissues of HCC patients.

	PFKFB3 High	PFKFB3 Low	
Clinicopathological Factors	(*N* = 43)	(*N *= 44)	*p*‐value
**Gender**			
female	9 (20.9%)	6 (13.6%)	.537
male	34 (79.1%)	38 (86.4%)	
**Age (years)**			
Mean (SD)	56.0 (12.6)	53.2 (12.7)	.294
**HBV**			
negative	10 (23.3%)	7 (15.9%)	.553
positive	33 (76.7%)	37 (84.1%)	
**TB (umol/L)**			
≤17.1	36 (83.7%)	37 (84.1%)	1.000
> 17.1	7 (16.3%)	7 (15.9%)	
**ALT (U/L)**			
Mean (SD)	39.7 (32.9)	37.7 (26.0)	.755
**PT (s)**			
Mean (SD)	12.7 (1.07)	13.1 (1.04)	.205
**Albumin (g/L)**			
Mean (SD)	3.87 (0.341)	3.96 (0.328)	
**AFP (ng/mL)**			
≤400	27 (62.8%)	29 (65.9%)	.936
> 400	16 (37.2%)	15 (34.1%)	
**Tumour size (cm)**			
Mean (SD)	7.53 (3.63)	5.57 (2.73)	**0.006**
**Tumour number**			
Mean (SD)	1.19 (0.450)	1.11 (0.387)	0.424
**Thrombi**			
No	21 (48.8%)	30 (68.2%)	.107
Yes	22 (51.2%)	14 (31.8%)	
**TNM**			
I‐II	30 (69.8%)	36 (81.8%)	.288
III	13 (30.2%)	8 (18.2%)	
**Histological grade**			
I‐II	33 (76.7%)	34 (77.3%)	1.000
III	10 (23.3%)	10 (22.7%)	
**COL1 expression**			
Mean (SD)	58.0 (11.6)	48.7 (18.0)	**.005**
**LOX expression**			
Mean (SD)	68.6 (9.48)	57.4 (18.3)	**<.001**
**COL1&LOX expression**			
COL1^High^/LOX^High^	18 (41.9%)	14 (31.8%)	**.008**
COL1^High^/LOX^Low^	8 (18.6%)	3 (6.8%)	
COL1^Low^/LOX^High^	8 (18.6%)	3 (6.8%)	
COL1^Low^/LOX^Low^	9 (20.9%)	24 (54.5%)	

Abbreviations: AFP, alpha‐fetoprotein; ALT, alanine aminotransferase; COL1, collagen 1; HBV, hepatitis B virus; HCC, hepatocellular carcinoma; LOX, lysyl oxidase; PFKFB3, phosphofructokinase‐2/fructose‐2,6‐bisphosphatase 3; PT, prothrombin time; TB, total bilirubin; TNM, tumour node metastasis.

### 2.2 A gel‐based culture system with tunable stiffness

FN‐coated polyacrylamide gel substrates with stiffness of 6 KPa (low‐stiffness substrate), 10 KPa (medium‐stiffness substrate) and 16 KPa (high‐stiffness substrate) were prepared for cell culture as the method described previously.^28^ Cell suspensions were seeded and spread on the top of the gel substrates, and incubated for 2∼3 h in a 37°C cell incubator. When cells were adherent to the gels completely, appropriate culture medium was added gently. Further steps were the same as the steps of normal cell culture.

### 2.3 SD rats HCC model with high liver stiffness background

All laboratory animals were cared and treated humanely according to the rules of the Institutional Animal Care and Use Committee of Zhongshan Hospital, Fudan University. SD rats HCC model with high liver stiffness background was established as the method described previously.^28^ Briefly, male SD rats (3∼4 weeks, Shanghai Slack Laboratory Animal Company) were subcutaneously injected with 100% carbon tetrachloride (3 mL/kg) in the abdomen in the first week. Starting from the second week, SD rats were injected with 50% carbon tetrachloride olive solution (2 mL/kg) twice a week (on Monday and Thursday). The intervention lasted for 12 weeks until SD rats with high liver stiffness were developed. Subsequently, the irradiated McA‐RH7777 cells (6 × 10^6^) and unirradiated McA‐RH7777 cells (6 × 10^6^) were resuspended in 100 µL 1 × PBS, respectively, and then they were gently mixed with 100 µL Matrigel (low growth factor, Corning). Cell mixtures were orthotopically injected under the liver capsule of rat, followed by injection with dexamethasone (6.5 mg/kg) intramuscularly on the first and second day before and after surgery, as well as on the day of the surgery.

On the basis of liver stiffness level and whether the inoculated HCC cells have been irradiated with X‐ray, the established HCC animal models with normal and high liver stiffness background were divided into four groups including normal liver stiffness group (group N), high liver stiffness group (group H), normal liver stiffness and irradiation group (group N+IR), and high liver stiffness and irradiation group (group H+IR). Seventeen days later, tumour tissues from these four groups were collected, and the length, short diameter and weight of the tumour were measured. Tumour volume was calculated according to the formula: 0.5 × length × (short diameter)^2^.

### 2.4 X‐ray radiation

HCC cells that were intended to receive X‐ray radiation were transported horizontally to the linear accelerator room. To correct the distribution of radiation, cell dishes were placed on a 1‐cm‐thick soft rubber pad, and then the cells were exposed to a dose of 2–14 Gy X‐ray radiation emitted by a linear accelerator (Oncor; Siemens). X‐ray radiation parameters were set as follows: 6MV‐X, 300MV/min dose rate, 180° gantry angle, and room temperature. Once the irradiation ended, the cells were immediately transferred to the cell culture incubator.

### 2.5 Lentiviral transduction of shRNA

Targeted gene sequence design, vector construction and virus packaging were completed by Shanghai Jikai Gene Co., Ltd. The corresponding double strands DNA fragments were subcloned into GV112 vector for PIEZO1 and Integrin β1 (ITGB1), and GV115 vector for PFKFB3 and NEDD4. Approximately 1 × 10^5^ HCC cells suspended in 1 mL complete medium were seeded into a 6‐well plate. When they grew to 30%–40% confluence, the cells were infected with lentivirus by sequentially adding complete medium, 40 µL Hitans GP transfection reagent, and viral stock solution. The multiplicity of infection (MOI) of LV‐shRNA‐ITGB1 was 5, and the others were 20. The efficiency of target gene knockdown was assessed by immunoblotting. The shRNA targeted sequences were as follows: (PIEZO1) ccCTGTGCATTGATTATCCCT, (ITGB1) CCTCCAGATGACATAGAAA, (PFKFB3) ctCCAATATCATGGAAGTTAA and (NEDD4) gcTGAACTATACGGTTCAAAT.

### 2.6 Intervention of Yoda1, GsMTx4, MG132, CHX and CQ

Yoda1 (Piezo1 agonist, MedChemExpress) was applied at the concentration of 5 µM for 48 h, while GsMTx4 (Piezo1 antagonist, Abcam) was used at 2.5 µM for 48 h. HCC cells were subjected to MG132 (a proteasome inhibitor, Cell Signaling Technology) at 10 µM for 6 h, which prevents PFKFB3 degradation via the proteasome pathway. Cycloheximide (CHX) (MedChemExpress) was used at 100 µg/mL and collected at the indicated times. Cycloheximide (CQ) (MedChemExpress) was added into cells by 10 µM for 6 h.

### 2.7 Extraction of nuclear protein

Nuclear protein extraction was performed according to the manufacture's instruction using the Nuclear and Cytoplasmic Extraction Kit (ThermoFisher). The cells were harvested freshly and rinsed using 1× PBS. Cell pellets were mixed with ice‐cold CER I and vortexed vigorously for 15 s, and then incubated on ice for 10 min. Subsequently, sample in the tube was mixed with ice‐cold CER II and then vortexed for 15 seconds, followed by incubating for 2 min on ice and centrifuging for 15 min at 16 000 g. After carefully removing the supernatants, the remnant fraction was washed using 1× PBS, and then mixed with ice‐cold NER. After continually vortexed for 40 min, nuclear extracts were obtained by centrifugation at 16 000 g for 10 min.

### 2.8 Neutral comet assays

Cells were collected and resuspended in PBS (1 × 10^5^/mL). LMAgarose (Trevigen) was melted and precooled to 37°C, and then aliquoted into an EP tube. Subsequently, 50 µL of above cell suspension was transferred into an EP tube and mixed with 500 µL LMAgarose. 50 µL of the mixture were pipetted and spread evenly onto the sample area of the CometSlide. The slides were placed in a 4°C wet box for 10 min, and immersed in lysis solution for 40 min at 4°C. Next, excess buffer was drained from slides, and then immediately immersed in 1× neutral electrophoresis buffer for 30 min at 4°C. For electrophoresis, set power at 21 volts. After precipitated by DNA precipitation solution for 30 min and fixed by 70% ethanol for 30 min at room temperature, the samples on the slides were stained using DAPI/PI (Beyotime), observed and photographed in a fluorescence microscope (Olympus). The images obtained were analysed by Casplab_1.2.3b2.

### 2.9 Plasmids and transient transfection

The plasmids encoding for human PFKFB3‐Vector, human PFKFB3‐WT, human PFKFB3‐E3A/Q10A/S25A, and human PFKFB3‐T339A/E343A/Q363A were purchased from Shanghai GeneChem, Co.Ltd. . The vector GV657 was used for construction of recombinant plasmid. The recombinant plasmids were transfected into HCC cells using Lipofectamine 3000, as the method described by the manufacturer (Invitrogen).

### 2.10 Molecular docking analysis

Molecular docking analyses were performed using the Schrödinger software. The crystal structures of two proteins, PFKFB3 (PDB entry 2AXN) and XRCC6 (PDB entry 1JEQ), were downloaded from the Protein Data Bank (PDB) database (https://www.rcsb.org/). The protein structure was optimized using protein preparation wizard module in Schrödinger. Then the structure of ligand was prepared in LigPrep. Docking was performed using Prime mode in Schrödinger, and binding free energies were calculated by the MM‐GBSA approach using the Prime MM‐GBSA module in Schrödinger software.

### 2.11 miRNA mimics and inhibitors

The hsa‐miR‐199a‐3p mimic and the control mimics (NC), as well as the hsa‐miR‐199a‐3p inhibitor and the control inhibitor (NC), were all purchased from RiboBio. Use the Lipofectamine RNAiMax kit (Invitrogen, USA) to transfect miRNA mimic and miRNA inhibitor according to the manufacturer's protocol. The sequences of miRNA mimic and inhibitor are as follows: mimic NC:UUUGUACUACACAAAAGUACUG; hsa‐miR‐199a‐3p mimic: ACAGUAGUCUGCACAUUGGUUA; inhibitor NC: CAGUACUUUUGUGUAGUACAAA; hsa‐miR‐199a‐3p inhibitor: UAACCAAUGUGCAGACUACUGU.

### 2.12 Seahorse analyses

Briefly, the cells were seeded at a density of 4 × 10^4^ per well. The following day media was replaced with glucose (10 mM) and then extracellular acidification rate (ECAR) were measured using a Seahorse 96XF instrument (Seahorse Bioscience, Agilent). Baseline measurements of ECAR were sequential supplemented with oligomycin (4 µM) and glycolytic inhibitor 2‐deoxyglucose (500 mM).

### 2.13 Statistical analyses

The statistical analyses were performed using GraphPad Prism 8.0 (GraphPad Software). All results are expressed as mean ± SD. To compare two group with continuous data, the two tailed, unpaired Student's *t*‐test was used. To compare two factors among multiple groups, two‐way ANOVA was applied. The Pearson chi‐square test was used for categorical data. A *p*‐value < .05 was considered statistically significant.

## 3 RESULTS

### 3.1 Matrix stiffness potentiates tumour growth and radiotherapeutic resistance in HCC

**FIGURE 1:**
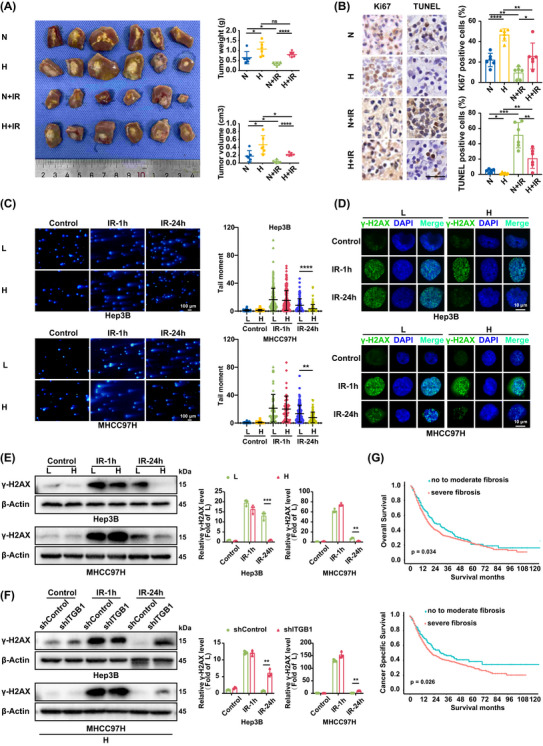
Matrix stiffness potentiates tumour growth and radiotherapeutic resistance in HCC. (A) SD rat HCC models with normal and high liver stiffness backgrounds were developed to clarify the effects of matrix stiffness on the growth of tumours from irradiated or unirradiated McA‐RH7777 cells. N, normal liver stiffness group; H, high liver stiffness group; N+IR, normal liver stiffness and irradiation group; H+IR, high liver stiffness and irradiation group. The weight and volume of tumours among different groups were comparatively analysed. (B) Left: Representative IHC images of Ki67 and TUNEL expression. Right: quantification of average percentage of Ki67 positive and TUNEL positive cells in tumour tissues in group N, group H, group N+IR, and group H+IR. Scale bar: 20 µm. (C) The tail moment of HCC cells on low‐stiffness and high‐stiffness substrates 1 h and 24 h after irradiation (Scale bar, 100 µm) in neutral comet assay and quantification of tail moment per cell (*n* > 50 cells). (D) Representative immunofluorescence images of γ‐H2AX foci in HCC cells on low‐stiffness and high‐stiffness substrates 1 h and 24 h after irradiation. Nuclear was stained with DAPI (blue). Scale bar, 10 µm. (E) Analysis of γ‐H2AX expression in HCC cells on low‐stiffness and high‐stiffness substrates 1 h and 24 h after irradiation. L, low‐stiffness substrate (6 kPa); H, high‐stiffness substrate (16 kPa). (F) Analysis of γ‐H2AX expression in HCC cells with integrin β1 knockdown on high‐stiffness substrates 1 h and 24 h after irradiation. H, high‐stiffness substrate (16 kPa). (G) Overall survival and cancer specific survival analysis of HCC patients received radiotherapy with different fibrosis stage from SEER database. IR, irradiation. shControl, empty vector; shITGB1, Integrin β1 knockdown. Values represent mean (SD); *p < .05, **p < .01, ***p < .001, ****p < .0001, ns: not significant; two‐tailed Student's *t*‐test.

SD rat HCC models with differential liver stiffness backgrounds were generated using a joint modelling method^28^ to investigate how matrix stiffness modulate the growth of tumours derived from unirradiated or irradiated HCC cells. As shown in the animal experiment flowchart (Figure S1A), SD rats with high liver stiffness were first induced by long‐term abdomen subcutaneous injection with CCL_4_. The liver matrix stiffness in the constructed model has been confirmed in article previously published by our group.^28^ Subsequently, the rats with high liver stiffness and the controls with normal liver stiffness were orthotopically inoculated with irradiated or unirradiated HCC cells (McA‐RH7777) suspended in Matrigel, followed by treatment with dexamethasone (as detailed in Methods and Materials). The optimal dose of irradiated McA‐RH7777 cells was set to 4 Gy, corresponding to 80% cell survival (Figure S1B). Seventeen days post‐inoculation, SD rat HCC models with differential liver stiffness backgrounds were successfully reconstituted. On the basis of liver stiffness level and whether the inoculated HCC cells have been irradiated with X‐ray, the established HCC animal models with normal and high liver stiffness backgrounds were divided into four groups including normal liver stiffness group (group N), high liver stiffness group (group H), normal liver stiffness and irradiation group (group N+IR), and high liver stiffness and irradiation group (group H+IR). Average weight and volume of HCC tumours in group H were all significantly higher than those in group N (Figure 1A), suggesting that HCC tumours in high stiffness liver grew faster than those in normal stiffness liver, in agreement with our previous findings.^29^ On the other hand, in both high and normal liver stiffness groups, the sizes of tumours from irradiated HCC cells were all prominently smaller than those from unirradiated HCC cells (Figure 1A). Simultaneously, the fold change of tumours weight and tumour volume from irradiated HCC cells in high liver stiffness group was higher than that from irradiated HCC cells in normal liver stiffness group (Figure 1A; Figure S1C), indicating that the growth of tumours from irridiated HCC cells in high liver stiffness group is superior to that in normal liver stiffness group. Furthermore, we examined the Ki67 and TUNEL in the four groups, showed that the expression level of Ki67 (a proliferation marker) in HCC tumours in group H was obviously elevated compared to those in group N (Figure 1B) and was increased in group H+IR compared to those in group N+IR. Consistently, TUNEL result was opposite correspondingly. Overall, these results illustrate that increased liver matrix stiffness can effectively promote tumour growth, also enhance cell survival from DNA damage to attenuate radiotherapeutic effect.

To validate the findings from animal models that matrix stiffness endowed cells with the ability of radiotherapy resistance, we prepared 6 KPa (L) and 16 KPa (H) gel substrates in vitro as described previously^29^, ^30^ to simulate tissue stiffness level of normal liver and cirrhotic liver,^30^ and analysed the effect of matrix stiffness on cell survival from DNA damage. According to the corresponding dose for 75% survival rate of cells in the dose‐survival curve (Figure S1D), we determined 4 Gy x‐ray as the optimal irradiation dose for subsequent cell tests. Substrate stiffness robustly promoted the survival of irradiated HCC cells, thereby indicating a direct role of biomechanical cues in radiotherapy efficacy (Figure S1E). Conversely, knockdown of integrin β1, a stiffness‐sensor molecule, clearly impaired the survival of the irradiated cells on high‐stiffness substrates (Figure S1E). These data indicate that matrix stiffness is indeed able to enhance the survival of the irradiated HCC cells. Neutral comet assay revealed that DNA damage in irradiated HCC cells was more rapidly and effectively repaired on high‐stiffness substrate. Although the irriadiated cells exhibited substantial tail moments immediately post‐irradiation(1 h), those under high stiffness stimulation displayed a significantly greater reduction in tail moment by 24 h, indicating superior repair efficiency (Figure 1C), in line with the findings in animal experiment. Meanwhile, the dynamics of DNA damage were analysed by monitoring γ‐H2AX foci formation and expression, a canonical DNA double strands break marker, in HCC cells on different stiffness substrates at 1 h and 24 h post‐irradiation. γ‐H2AX foci and γ‐H2AX expression level in HCC cells were all remarkably improved in high‐ and low‐stiffness substrate groups at the 1st hour after irradiation. However, at the 24th hour after irradiation, γ‐H2AX foci and γ‐H2AX expression level in HCC cells on high‐stiffness substrates were all dramatically decreased, compared with those on low‐stiffness substrates. (Figure 1D,E; Figure S1F). The above results were the same as the findings of neutral comet assay, reconfirming that high stiffness stimulation can significantly improve the DNA damage repair ability of HCC cells. Besides, the promoting effect of matrix stiffness on DNA damage repair was found to be dependent on integrin β1, as its knockdown markedly impaired the repair ability in cells on high‐stiffness substrates (Figure 1F). Collectively, matrix stiffness as an initial contributor can effectively enhance cell survival from DNA damage, and participate in radiotherapeutic resistance of HCC.

Furthermore, we collected clinical data of HCC patients from SEER database (Figure S1G) and evaluated the effect of different fibrosis stages on radiotherapy resistance. Based on the classification method of Ishak et al.,^31^ we divided HCC patients who underwent radiotherapy into group Ishak 0–4 and group Ishak 5–6. Group Ishak 0–4 was defined as no to moderate fibrosis group, and group Ishak 5–6 was defined as advanced to severe fibrosis group. The overall survival for patients received radiotherapy in group Ishak 0–4 was significantly longer than that in group Ishak 5–6 (median: 23 vs. 17 months; *p* = 0.034) (Figure 1G ). Similarly, the cancer‐specific survival for patients received radiotherapy in group Ishak 0–4 was significantly longer than that in group Ishak 5–6 (median: 31 vs. 21 months; *p* = 0.026) (Figure 1G ). These clinical data also indirectly indicate that matrix stiffness significantly attenuate radiotherapeutic effect, supporting the findings in animal experiment and cell test in vitro. Univariate and multivariate analyses identified independent predictors of overall survival (OS, Table S1) and cancer‐specific survival (CSS, Table S2) in HCC. Multivariate analysis demonstrated that for OS, an Ishak fibrosis score of 0–4 was associated with a decreased risk of death compared to a score of 5–6 (HR = 0.73, 95% CI: 0.60–0.90, *p* = 0.003), and an Ishak fibrosis score of 0–4 was associated with a decreased risk of CSS event compared to a score of 5–6 (HR = 0.70, 95% CI: 0.56–0.88, *p* = 0.002).

**FIGURE 2:**
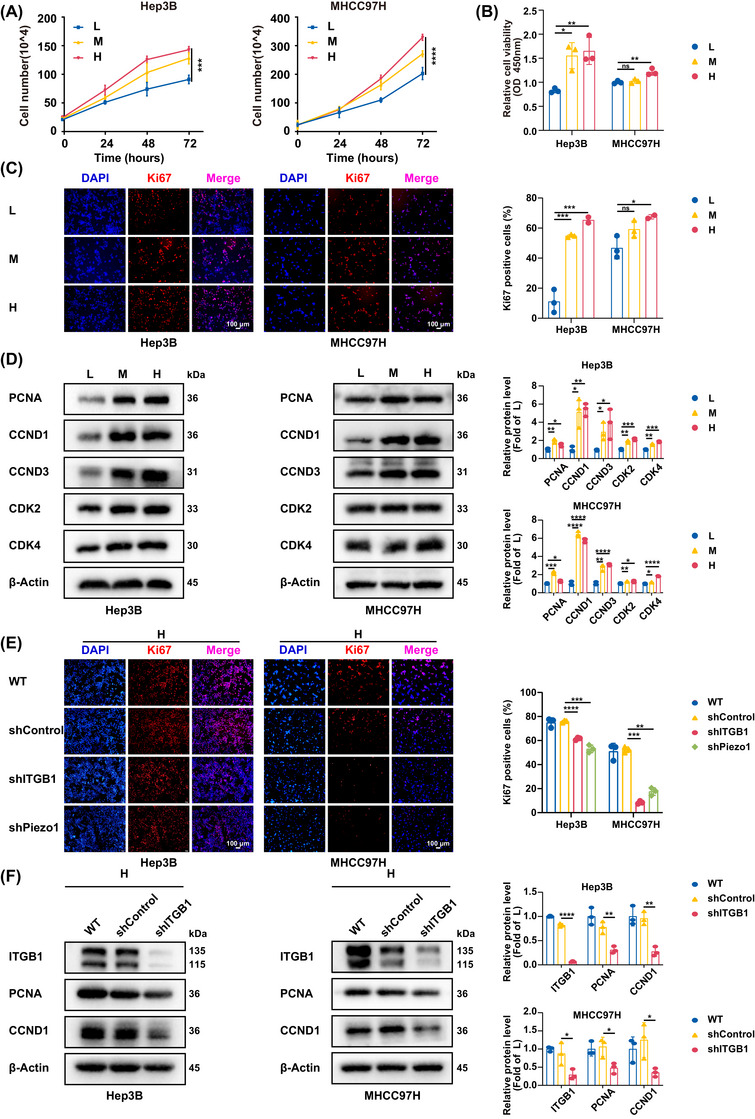
High matrix stiffness promotes cell proliferation in HCC. (A) Analysis of the growth of HCC cells on different stiffness substrates (L, 6 kPa; M, 10 kPa; H, 16 kPa) at indicated times. (B) Cell viability of HCC cells on different stiffness substrates (L, 6 kPa; M, 10 kPa; H, 16 kPa) for 72 h, detected by CCK‐8 assay. (C) Representative immunofluorescence images of Ki67 expression in HCC cells on different stiffness substrates (L, 6 kPa; M, 10 kPa; H, 16 kPa) and quantification of percentage of Ki67 positive cells per image. Nuclear was stained by DAPI. Scale bar, 100 µm. (D) Expressions of proliferation‐associated proteins in HCC cells on different stiffness substrates (L, 6 kPa; M, 10 kPa; H, 16 kPa). PCNA, proliferating cell nuclear antigen; CCND1, cyclin D1; CCND3, Cyclin D3; CDK2, cyclin‐dependent kinase K2; CDK4, cyclin‐dependent kinase K4. (E) Representative immunofluorescence images of Ki67 expression in HCC cells with shITGB1 or shPiezo1 on high‐stiffness substrate and quantification of percentage of Ki67 positive cells per image. Nuclear was stained by DAPI. Scale bar, 100 µm. (F) Suppression of integrin β1 significantly downregulated PCNA and CCND1 expressions in HCC cells on high‐stiffness substrate. L, low‐stiffness substrate (6 kPa); M, medium‐stiffness substrate (10 kPa); H, high‐stiffness substrate (16 kPa). WT, wild type; shControl, empty vector; shITGB1, Integrin β1 knockdown; shPiezo1, Piezo1 knockdown. Values represent mean (SD); *p < .05, **p < .01, ***p < .001, ****p < .0001, ns: not significant; statistical significance was analysed using two‐way ANOVA analysis (A) or two‐tailed Student's *t*‐test (except A).

Additionally, we compared the growth of HCC cells on different stiffness substrates and found that the proliferation rate and the viability of HCC cells and their Ki67 expression were all distinctly improved as matrix stiffness increased (Figure 2A–C). Similarly, proliferation‐associated proteins such as proliferating cell nuclear antigen (PCNA), cyclin D1 (CCND1), Cyclin D3 (CCND3), cyclin‐dependent kinase K2 (CDK2), and cyclin‐dependent kinase K4 (CDK4) also exhibited an obvious increasing trend in the expression level (Figure 2D). These results convincingly supported the promoting effect of matrix stiffness on cell proliferation, in accordance with the results of animal experiment. On the contrary, suppression of stiffness‐sensor molecule (integrin β1 or Piezo1) significantly downregulated Ki67 (Figure 2E), PCNA and CCND1 expressions (Figure 2F; Figure S2) in cells on high‐stiffness substrate, also reversely testifying the contribution of high stiffness stimulation on cell proliferation in HCC.

### 3.2 PFKFB3 drives stiffness‐dependent HCC proliferation and radiotherapeutic resistance

**FIGURE 3:**
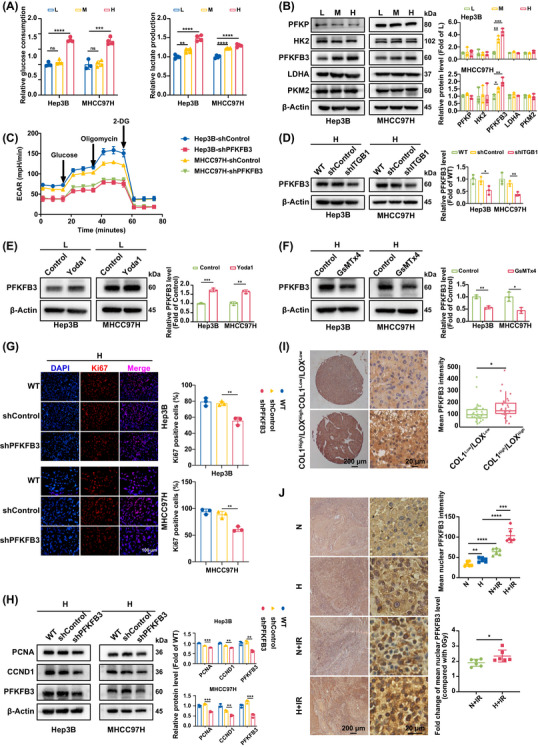
Metabolic enzyme PFKFB3 participates in matrix stiffness‐mediated effect on proliferation and radiotherapeutic resistance in HCC. (A) Comparative analysis of glucose consumption and lactate production in HCC cells on different stiffness substrates (L, 6 kPa; M, 10 kPa; H, 16 kPa), their relative contents were normalized to cell number. (B) Analysis of glycolytic enzyme expressions in HCC cells on different stiffness substrates (L, 6 kPa; M, 10 kPa; H, 16 kPa). PFKP, phosphofructokinase, platelet; HK2, hexokinase 2; PFKFB3, phosphofructokinase‐2/fructose‐2,6‐bisphosphatase 3; LDHA, lactate dehydrogenase A; PKM2, pyruvate kinases M2. (C) Evaluation of the glycolysis rate of FPKFB3 knockdown cells as measured by ECAR. (D) Analysis of PFKFB3 expression in HCC cells with shITGB1 on high‐stiffness substrate. (E) The expression of PFKFB3 in HCC cells on low‐stiffness substrate after intervention with Yoda1 (5 µM) for 48 h. (F) The expression of PFKFB3 in HCC cells on high‐stiffness substrate after intervention with GsMTx4 (2.5 µM) for 48 h. (G) Representative immunofluorescence images of Ki67 expression in HCC cells with shPFKFB3 on high‐stiffness substrate and quantification of percentage of Ki67 positive cells per image. Nuclear was stained by DAPI. Scale bar, 100 µm. (H) Expressions of proliferation‐associated proteins (PCNA, CCND1) in HCC cells with shPFKFB3 on high‐stiffness substrate. (I) The expression level of PFKFB3 in human HCC tissues in COL1^High^/LOX^High^ group (high‐stiffness group, 32 cases) was significantly higher than that in COL1^Low^/LOX^Low^ group (low‐stiffness group, 33 cases). Scale bar: left, 200 µm; right, 20 µm. (J) Analysis of the expression and distribution of PFKFB3 in rat HCC tissues from group N, group H, group N+IR, and group H+IR. Scale bar: left, 200 µm; right, 20 µm. Right up panel, quantification of mean nuclear PFKFB3 intensity. Right down panel, Fold change of mean nuclear PFKFB3 intensity in rat HCC tissues from irradiation group with normal or high liver background, compared with corresponding unirradiated group. N, normal liver stiffness group; H, high liver stiffness group; N+IR, normal liver stiffness and irradiation group; H+IR, high liver stiffness and irradiation group. L, low‐stiffness substrate (6 kPa); M, medium‐stiffness substrate (10 kPa); H, high‐stiffness substrate (16 kPa). shControl, empty vector; WT, wild type; shITGB1, Integrin β1 knockdown; shPFKFB3, PFKFB3 knockdown. Values represent mean (SD); *p < .05, **p < .01, ***p < .001, ****p < .0001, ns: not significant; two‐tailed Student's *t*‐test.

Considering that the prominent role of glycolytic enzymes in proliferation and survival of tumour cells^32^ and their non metabolic functions,^27^ we speculated that glycolytic enzymes might mediate matrix stiffness‐potentiated proliferation and radiotherapeutic resistance. This phenotype was confirmed by significantly elevated levels of glucose consumption and lactate production in cells on stiff substrates (Figure 3A). Simultaneously, knockdown of integrin β1 or Piezo1 distinctly attenuated high stiffness stimulation‐caused glucose consumption and lactate production (Figure S3A), indicating that matrix stiffness enhances glycolytic level of HCC cells, and thereby promotes tumour growth and proliferation. Given that glycolytic flux is often reflected by the abundance of key enzymes,^25^, ^26^ we hypothesized that increased matrix stiffness might regulate the expressions of metabolic enzymes. Profiling key glycolytic enzymes in HCC cells under different stiffness stimulation revealed a striking, selective upregulation of PFKFB3, but other critical glycolytic enzymes, including phosphofructokinase platelet (PFKP), hexokinase 2 (HK2), lactate dehydrogenase A (LDHA) and pyruvate kinases M2 (PKM2), remained unaltered in expression (Figure 3B). Further, PFKFB3 knockdown decreased the extracellular acidification rate (ECAR) (an indicator of glycolysis) in HCC cells (Figure 3C), implying that increased matrix stiffness strengthens glycolytic level of HCC cells mainly through upregulating PFKFB3 expression. The stiffness‐induced PFKFB3 upregulation was demonstrated to depend on integrin β1 and Piezo1 signalling. Genetic silencing of either component abrogated the PFKFB3 upregulation on high‐stiffness substrate (Figure 3D; Figure S3B). Furthermore, pharmacological activation of Piezo1 with Yoda1 sufficed to elevate PFKFB3 expression in HCC cells on low‐stiffness substrate, while its inhibition with GsMTx4 on high‐stiffness substrate prevented its upregulation (Figure 3E,F). The data above strongly indicate that high stiffness stimulation upregulates PFKFB3 expression via stiffness‐sensor molecule integrin β1 or Piezo1. We further examined the changes of proliferation indexes in HCC cells with PFKFB3 knockdown under high‐stiffness stimulation, and found that PFKFB3 suppression significantly downregulated the expressions of Ki67 (Figure 3G) and proliferation‐associated proteins PCNA, and CCND1 (Figure 3H) in HCC cells, confirming a positive correlation between PFKFB3 expression and cell proliferation. Additionally, PFKFB3 expression was significantly higher in human HCC tissues classified as high‐stiffness (COL1^High^/LOX^High^, *n* = 32) versus low‐stiffness (COL1^Low^/LOX^Low^, *n *= 33) (Figure 3I), and its pathological significance analysis also supported a positive association between high PFKFB3 expression and increased tumour size (Table 1). Overall, PFKFB3, an indirect rate‐limiting enzyme for glycolysis,^33^ mediates matrix stiffness‐promoted tumour growth and proliferation.

**FIGURE 4:**
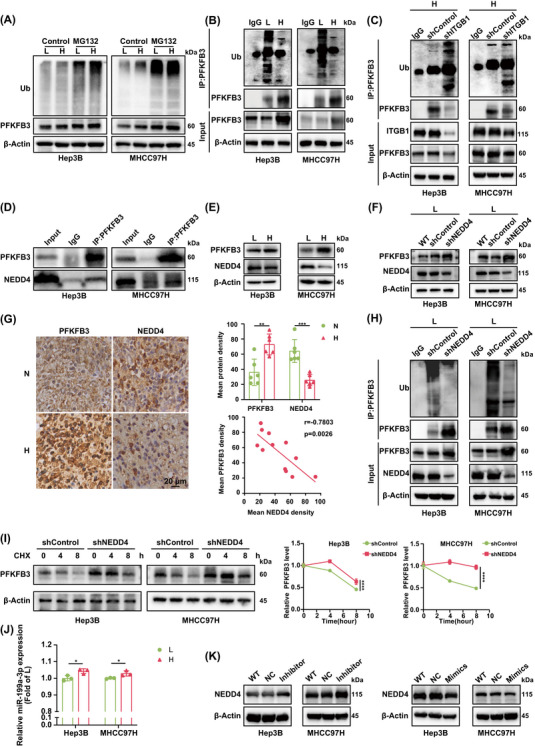
High stiffness stimulation significantly improves PFKFB3 expression in HCC cells by lessening its ubiquitination level. (A) Expressions of PFKFB3 and ubiquitination in HCC cells on low‐stiffness and high‐stiffness substrates under intervention of MG132 (10 µM) for 6 h. Control, DMSO. L, low‐stiffness substrate (6 kPa), and H, high‐stiffness substrate (16 kPa). (B) Abundance of polyubiquitinated PFKFB3 upon PFKFB3 pull‐down using whole cell protein of HCC cells on low‐stiffness and high‐stiffness substrates. (L, 6 kPa; H, 16 kPa). (C) Abundance of polyubiquitinated PFKFB3 upon PFKFB3 pull‐down using whole cell protein of HCC cells with shITGB1 on high‐stiffness substrates. (H, 16 kPa). (D) Co‐IP combining immunoblotting validated that there was an interaction between NEDD4 and PFKFB3. (E) Expressions of PFKFB3 and NEDD4 in HCC cells on low‐stiffness and high‐stiffness substrates. NEDD4, neural precursor cell expressed, developmentally down‐regulated 4. (L, 6 kPa; H, 16 kPa). (F) Abundance of PFKFB3 in shNEDD4 HCC cells on low‐stiffness substrate. (L, 6 kPa). (G) Representative IHC images of PFKFB3 and NEDD4 expression in rat HCC tissues with normal and high liver stiffness backgrounds (left panel). Scale bar, 20 µm. Quantification of mean PFKFB3 and NEDD4 density (right up panel). Values represent mean (SD); two‐tailed Student's *t*‐test. **p < .01, ***p < .001. Correlation analysis of PFKFB3 and NEDD4 expression levels (right down panel), using the Pearson correlation coefficient. N, normal liver stiffness group; H, high liver stiffness group. (H) Abundance of polyubiquitinated PFKFB3 upon PFKFB3 pull‐down using whole cell protein of HCC cells with shNEDD4 on low‐stiffness substrate. (I) The degradation of PFKFB3 in HCC cells with shNEDD4. Cells were intervened with CHX (100 µg/mL) and collected at the indicated times. Two‐way ANOVA, ****p < .0001. (J) The relative miR‐199a‐3p expression in HCC cells on different stiffness substrates. two‐tailed Student's *t*‐test. *p < .05. (K) Abundance of PFKFB3 in HCC cells treated with miR‐199a‐3p inhibitor or mimics. Ub, ubiquitin; IgG, isotype control; shControl, empty vector; shITGB1, Integrin β1 knockdown; shNEDD4, NEDD4 knockdown.

In addition to possessing the classic functions of glucose metabolism, metabolic enzymes can also exert non‐metabolic functions to modulate cellular activities and disease progression through nuclear translocation.^27^, ^34^ So, we reasoned that glycolytic enzyme PFKFB3 might undergo nuclear translocation to participate in matrix stiffness‐mediated effect on radiotherapeutic resistance in HCC. We comparatively analysed the expression and distribution of PFKFB3 in HCC tissues from group N, group H, group N+IR, and group H+IR. Same as the in vitro findings, PFKFB3 levels were discernibly heightened in HCC tissues from the high liver stiffness group compared to the normal stiffness group (Figure 4G). Meanwhile, PFKFB3 in HCC tissues in normal liver stiffness group mainly distributed in the cytoplasm of tumour cells, but in high liver stiffness group, PFKFB3 expression occurred in both the cytoplasm and the nucleus (Figure 3J). More importantly, nuclear expression of PFKFB3 in group H+IR was much higher than that of groups H and N+IR. It exhibits an obvious increase in nuclear translocation of PFKFB3 in the irradiated HCC cells in high liver stiffness group (Figure 3J).

These findings indicate that the promotion of radiation resistance by high‐stiffness matrix is mediated, at least in part, through the induction of PFKFB3 nuclear translocation in HCC cells.

### 3.3 High stiffness stimulation significantly improves PFKFB3 protein expression in HCC cells by lessening its ubiquitination level

High stiffness stimulation prominently increased PFKFB3 expression in HCC cells at protein level (Figure 3B), but it failed to alter the mRNA expression of PFKFB3 (Figure S4A). This finding implicates that matrix stiffness‐caused PFKFB3 upregulation may be attributed to its ubiquitination modification. Inhibition of the proteasome with MG132 markedly augmented both PFKFB3 protein expression and overall ubiquitin abundance in HCC cells cultured on either high‐ or low‐stiffness substrate (Figure 4A; Figure S4B). Next, analysis of PFKFB3 pull down showed an obvious decrease in the ubiquitination level of PFKFB3 protein in HCC cells on high‐stiffness substrate (Figure 4B). Besides, Cycloheximide (CHX) chase assays confirmed that the degradation of PFKFB3 was slowed by high stiffness stimulation (Figure S4C,D), suggesting that high stiffness stimulation indeed impedes PFKFB3 ubiquitination, and in turn stabilize PFKFB3 protein expression. In addition, chloroquine (CQ) did not obviously rescue PFKFB3 expression in HCC cells on low‐stiffness substrate (Figure S4E), ruling out the role of lysosomal protein hydrolysis pathways in matrix stiffness‐modulated PFKFB3 expression. Conversely, suppression of integrin β1 and Piezo1 all evidently improved ubiquitination level of PFKFB3 protein and reduced the protein expression of PFKFB3 in HCC cells on high‐stiffness substrate (Figure 4C; Figure S4F), further confirming the role of matrix stiffness in PFKFB3 ubiquitination. Therefore, matrix stiffness upregulates PFKFB3 protein expression by inhibiting ubiquitin‐mediated protein degradation.

E3 ubiquitin ligase, which binds ubiquitin to the target protein,^35^ plays an important role in proteasome‐mediated protein degradation. Reducing the activity and expression of E3 ligase usually inhibits ubiquitination of the target protein, and thereby preventing the degradation of the target protein. To screen the E3 ubiquitin ligase of PFKFB3, we applied immunoprecipitation combined with LC‐MS/MS to analyse interaction proteins of PFKFB3 in HCC cells. Among the identified 2126 proteins, we obtained 15 common candidate proteins by comparing the human E3 ubiquitin ligase molecular set from the public database NHLBI (https://esbl.nhlbi.nih.gov/Databases/KSBP2/Targets/Lists/E3‐ligases/). Taking peptide spectrum matches (PSMs ) > 4 as the threshold, we further got 4 candidate E3 ligases targeted PFKFB3 including peptidylprolyl isomerase like 2 (PPIL2), pre‐mRNA processing factor 19 (PRPF19), E3 ubiquitin ligase tripartite motif (TRIM)‐containing protein 21 (TRIM21), and NEDD4 (Table S3). Due to low specificity, candidate proteins PPIL2 and TRIM21 with the ratios of peptides P3/IgG ≤ 1 were excluded. Simultaneously, using Kaplan‐Meier Plotter website in database‐liver hepatocellular carcinoma (LIHC) (http://kmplot.com/ analysis/index.php?p = service&cancer = liver_rnaseq), we evaluated the prognosis of the above candidate molecules in HCC patients, and found that only NEDD4 overexpression indicated favourable prognosis (Figure S4G). Based on the above results, it is easy to conclude that NEDD4 is likely to be the specific E3 ligase targeted PFKFB3. Subsequently, Co‐IP analysis also validated that there existed the endogenous interaction of PFKFB3 and NEDD4 in HCC cells (Figure 4D). Profiling of NEDD4 and PFKFB3 revealed stiffness‐dependent expression patterns. NEDD4 expression was significantly downregulated in HCC cells on high‐stiffness substrate, contrary to the upregulation trend of PFKFB3 expression (Figure 4E; Figure S4H). Importantly, suppression of NEDD4 substantially enhanced PFKFB3 protein expression (Figure 4F; Figure S4I) but failed to alter its mRNA expression (Figure S4J) on low‐stiffness substrate, further suggesting that NEDD4 is indeed a specific E3 ligase responsible for matrix stiffness‐caused PFKFB3 upregulation. Analysis of the SD rat HCC model revealed a marked negative correlation between NEDD4 and PFKFB3 expression, and HCC tissues in the high liver stiffness group were characterized by a distinct molecular signature of elevated PFKFB3 and suppressed NEDD4 (Figure 4G), also corroborating our in vitro data. Additionally, NEDD4 downregulation obviously inhibited PFKFB3 polyubiquitination and slowed degradation of PFKFB3 protein (Figure 4H,I). Taken together, high stiffness stimulation significantly upregulates PFKFB3 protein expression in HCC cells by reducing its ubiquitination level.

Additionally, integrin β1 or Piezo1 downregulation also reversed the expression of NEDD4 in HCC cells on high‐stiffness substrate (Figure S5A,B). Thus, we further investigated how matrix stiffness regulated NEDD4 expression. Experiments have showed that miR‐199a‐3p can decrease NEDD4 expression,^36^ and miR‐199a‐3p is positively related to mechanical stimulus.^37^ So, we hypothesized that miR‐199a‐3p may participate in matrix stiffness‐modulated NEDD4. We observed that miR‐199a‐3p expression was increased in HCC cells on high‐stiffness substrate (Figure 4J), but decreased in HCC cells with integrin β1 or Piezo1 knockdown on high‐stiffness substrate (Figure S5C). Furthermore, miR‐199a‐3p inhibitor strengthened NEDD4 protein level, while miR‐199a‐3p mimics inhibited NEDD4 protein level (Figure 4K; Figure S5D). So increased matrix stiffness downregulated NEDD4 expression via improving miR‐199a‐3p expression.

### 3.4 Nuclear translocation of PFKFB3 contributed to matrix stiffness‐induced DNA repair in HCC

**FIGURE 5:**
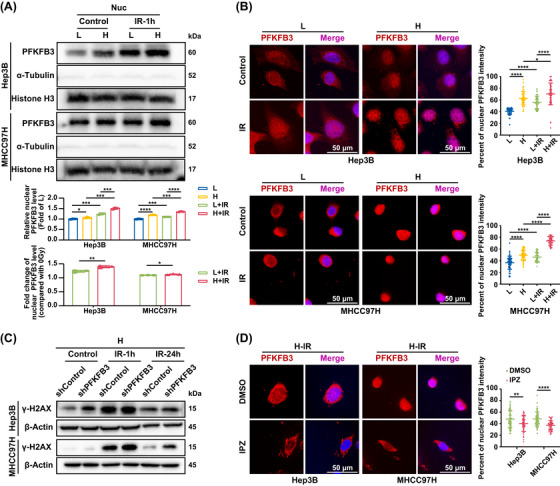
Nuclear translocation of PFKFB3 contributes to matrix stiffness‐induced DNA repair in HCC cells. (A) Up, abundance of PFKFB3 in the nuclear fraction of HCC cells on low‐stiffness and high‐stiffness substrates after irradiation for 1 h. Nuc, nuclear. Middle, quantification of relative nuclear PFKFB3, normalized to the abundance of Histone H3. Down, fold change of nuclear PFKFB3 level in HCC cells on low‐stiffness and high‐stiffness substrates after irradiation for 1 h, compared with unirradiated HCC cells. (B) Representative immunofluorescence images of PFKFB3 in HCC cells on low‐stiffness and high‐stiffness substrates after irradiation for 1 h, and quantification of percentage of nuclear PFKFB3 intensity per cell (n≥50 cells). Scale bar, 50 µm. (C) γ‐H2AX expression in HCC cells with PFKFB3 knockdown on high‐stiffness substrate 1 h and 24 h after irradiation. (D) Importazole intervention (20 µM) partially attenuated PFKFB3 nuclear translocation in the irradiated cells on high‐stiffness substrate. Scale bar, 50 µm. L, low‐stiffness substrate (6 kPa); H, high‐stiffness substrate (16 kPa). IPZ, importazole. Values represent mean (SD). *p < .05, **p < .01, ***p < .001, ****p < .0001; two‐tailed Student's *t*‐test.

According to the above results of PFKFB3 distribution in HCC tissues from animal models in group N, group H, group N+IR, and group H+IR (Figure 3J), we inferred that PFKFB3 nuclear translocation may be involved in matrix stiffness promoting radiation resistance in HCC. We first investigated whether matrix stiffness manipulated PFKFB3 nuclear translocation and associated with DNA damage repair. We respectively extracted nuclear proteins of the irradiated HCC cells on high‐ and low‐stiffness substrates to detect their PFKFB3 expression. The results showed that there existed PFKFB3 nuclear translocation in the irradiated HCC cells both on high‐ and low‐stiffness substrates (Figure 5A). Moreover, the irradiated cells on high‐stiffness substrate exhibited an obvious increase in nuclear accumulation of PFKFB3 compared to those on low‐stiffness substrate (Figure 5A). Consistently, immunofluorescence staining analysis also presented a similar result (Figure 5B), supporting the results of PFKFB3 nuclear translocation in animal experiment. Accordingly, high stiffness stimulation prominently increases PFKFB3 nuclear translocation in the irradiated HCC cells.

Subsequently, we further testified the association between PFKFB3 nuclear translocation and DNA damage repair. We suppressed PFKFB3 expression to examine survival of the irradiated HCC cells on high‐stiffness substrate, and discovered that PFKFB3 knockdown not only resulted in an obvious decrease in the survival of the irradiated cells on high‐stiffness substrate (Figure S6A), but also impeded DNA damage repair in the irradiated cells on high‐stiffness substrate (Figure 5C; Figure S6B). These results support that PFKFB3 mediates high matrix stiffness‐induced DNA repair. Given that nuclear translocation of PFKFB3 was mainly through the importin α/β pathway,^38^ we employed importazole, the importin β inhibitor, to intervene the irradiated HCC cells on high‐stiffness substrate, and immunofluorescence staining and nuclear protein analysis all demonstrated that importazole intervention partially attenuated PFKFB3 nuclear translocation in the irradiated cells on high‐stiffness substrate (Figure 5D; Figure S6C). Except that, importazole intervention suppressed DNA damage repair ability of the irradiated cells on high‐stiffness substrate (Figure S6D). In addition, 3PO (an inhibitor of PFKFB3) impaired glucose consumption and lactate production of cells on high‐stiffness substrate (Figure S6E). Nonetheless, 3PO failed to weaken DNA repair ability of HCC cells on high‐stiffness substate (Figure S6F).

Taken together, nuclear translocation of PFKFB3 was indeed crucial to matrix stiffness‐regulated DNA damage repair.

### 3.5 Nuclear PFKFB3 interacted with Ku70 to participate in DNA damage repair

**FIGURE 6:**
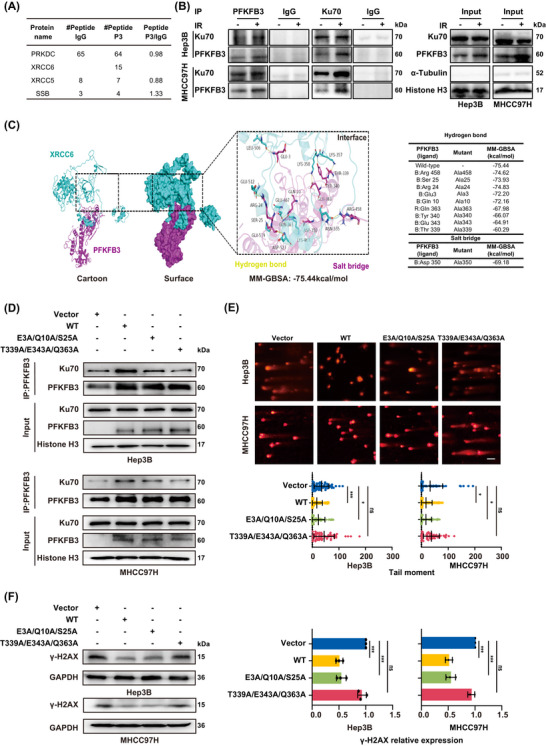
PFKFB3 interacts with Ku70 to participate in DNA damage repair. (A) The candidate DNA repair proteins binding to PFKFB3 in irradiated HCC cells identified by IP‐MS. IgG, Isotype control antibody; P3, PFKFB3 antibody. (B) Immunoblot of anti‐PFKFB3 or anti‐Ku70 immunoprecipitation from nuclear protein of HCC cells 2 h after irradiation. (C) Left, the molecular docking analysis of human PFKFB3 protein (purple, PDB entry 2AXN) and human XRCC6 protein (cyan, PDB entry 1JEQ) using Schrödinger software. Right, the MM‐GBSA changes after the non‐covalent interaction sites (hydrogen bonds and salt bridges) of PFKFB3 in the docking model are mutated to alanine. (D) Co‐IP assays displayed the interactions between Ku70 and PFKFB3 in HCC cells with different PFKFB3 recombinant plasmids (PFKFB3‐Vector, PFKFB3‐WT, PFKFB3‐E3A/Q10A/S25A, and PFKFB3‐T339A/E343A/Q363A). (E) Analysis of DNA damage levels in HCC cells with different recombinant plasmids (PFKFB3‐Vector, PFKFB3‐WT, PFKFB3‐E3A/Q10A/S25A, and PFKFB3‐T339A/E343A/Q363A) 24 h after irradiation using neutral comet assay and quantification of tail moment per cell (*n* > 50 cells). Scale bar: 100 µm. (F) γ‐H2AX expression in HCC cells with different PFKFB3 recombinant plasmids (PFKFB3‐Vector, PFKFB3‐WT, PFKFB3‐E3A/Q10A/S25A, and PFKFB3‐T339A/E343A/Q363A) 24 h after irradiation. Values represent mean (SD). *p < .05, ***p < .001, ****p < .0001, ns: not significant; two‐tailed Student's *t*‐test.

To shed light on the underlying mechanism of PFKFB3 nuclear translocation participating in DNA damage repair, we further screened and identified the potential binding proteins of PFKFB3 in nuclear protein from the irradiated HCC cells using immunoprecipitation combined with mass spectrometry. Taking PSM > 4 as the threshold, a total of 1005 nuclear binding proteins with PFKFB3 were obtained. By comparing the homologous recombination (HR) and non‐homologous end joining (NHEJ) pathway molecular sets from the public data analysis website KEGG, 4 common candidate proteins such as PRKDC, XRCC5, SSB and XRCC6 were determined (Figure 6A). Referring to the results of negative control antibody IgG, PRKDC, XRCC5, and SSB (Peptides P3/IgG are 0.98, 0.88, and 1.33, respectively) were likely to be non‐specific binding protein. Due to barely detectable of XRCC6 in the negative control IgG group, XRCC6 (also named as Ku70) may be the only protein interacting with PFKFB3 in the irradiated HCC cells. Subsequently, regardless of taking PFKFB3 as a bait protein to capture Ku70 or taking Ku70 as a bait protein to capture PFKFB3 in nuclear protein of irradiated HCC cells, we all verified an endogenous binding between PFKFB3, Ku70 and x‐ray repair cross‐complementing protein 4 (XRCC4) in the nucleus of irradiated HCC cells (Figure 6B; Figure S7A), suggesting that PFKFB3 may participates in NHEJ. Moreover, the interaction of two proteins was found to be enhanced after irradiation (Figure 6B).

To further clarify whether PFKFB3 interacting with Ku70 is indispensable for DNA damage repair, we applied Schrödinger software to analyse the macromolecular docking state and binding site region between PFKFB3 and Ku70. The molecular docking analysis showed that there was a direct physical binding between PFKFB3 and Ku70 (Figure 6C; Figure S7B). Once the interaction sites (Gln363, Tyr340, Glu343, Thr339) of PFKFB3 in the docking model were mutated to alanine, their corresponding MM‐GBSA energy was decreased obviously compared with that for other site mutations (Arg458, Ser25, Arg24, Glu3, Gln10). Thus, we respectively constructed two mutant plasmids of PFKFB3 (PFKFB3‐T339A/E343A/Q363A and PFKFB3‐E3A/Q10A/S25A) to determine the role of PFKFB3 interacting with Ku70 in DNA repair. The results unveiled that PFKFB3‐T339A/E343A/Q363A mutations sharply reduced interaction with Ku70 in nucleus of HCC cells (Figure 6D). On the other hand, PFKFB3‐WT and PFKFB3‐E3A/Q10A/S25A mutation obviously reduced tail moment (Figure 6E) and γ‐H2AX expression (Figure 6F), but PFKFB3‐T339A/E343A/Q363A mutation failed to decrease tail moment (Figure 6E) and γ‐H2AX expression (Figure 6F) at the 24th hour after irradiation in HCC cells compared with PFKFB3‐vector, indicating T339A/E343A/ Q363A mutation hindered the binding between PFKFB3 and Ku70, and lost the ability to protect cells from IR‐induced DNA damage. Thereby, PFKFB3 interacting with Ku70 was required for DNA damage repair.

### 3.6 Clinical significance of PFKFB3 in HCC patients

To corroborate the above findings in vivo and in vitro, we analysed PFKFB3 expression in tumour tissues from patients with HCC and explored its clinical significance. Taking the median expression values of collagen 1 (COL1) and LOX in HCC tissues as the threshold, we classified HCC tissues into high‐stiffness group (COL1^High^/LOX^High^, 32 cases) and low‐stiffness group (COL1^Low^/LOX^Low^, 33 cases). A stark contrast in PFKFB3 expression was observed, with levels being appreciably heightened in the high‐stiffness group (Figure 3I). Using the median PFKFB3 expression level as a cutoff, HCC patients were stratified into PFKFB3^high^ (*n* = 43) and PFKFB3^low^ (*n* = 44) cohorts for subsequent clinicopathological analysis. The results revealed that high PFKFB3 expression was positively correlated with larger tumour size, as well as increased expression of COL1, LOX and the COL1^High^/LOX^High^ signature (Table 1). These clinical findings directly linked elevated PFKFB3 expression to both tumour progression and a high‐stiffness microenvironment, in agreement with the findings in vivo and in vitro. Moreover, high expression of PFKFB3 indicated unfavourable prognosis in HCC patients, including overall survival (OS) and disease‐free survival (DFS) (Figure S8). Univariate and multivariate analysis revealed that high expression of PFKFB3 was an independent risk factor for OS (Table 2) and DFS in HCC patients (Table 3).

**TABLE 2:** Univariate and multivariate COX regression analysis of clinicopathological features associated with overall survival in HCC patients.

	Overall survival			
	Univariate		Multivariate	
Clinicopathlogical Factors	*p*‐value	HR (95%CI)	*p*‐value	HR (95%CI)
Gender: female vs male	**0.015**	2.3 (1.2‐4.5)	**0.018**	2.3 (1.2‐4.5)
Age(years): >50 vs ≤50	0.100	1.6 (0.91‐2.9)	NA	
HBV infection: Positive vs Negative	0.520	1.3 (0.6‐2.8)	NA	
TB(umol/L): >17.1 vs ≤17.1	0.840	0.92 (0.41‐2.1)	NA	
ALT(U/L): >40 vs ≤40	0.470	0.79 (0.41‐1.5)	NA	
PT(s): >14 vs ≤14	0.660	1.2 (0.51‐2.9)	NA	
AFP(ng/mL): >400 vs ≤400	0.280	1.4 (0.76‐2.5)	NA	
Tumor size(cm): >5 vs ≤5	**0.004**	2.5 (1.3‐4.6)	0.680	1.2 (0.56‐2.5)
Tumor number: Multiple vs Solitary	**0.089**	1.9 (0.9‐4.2)	NA	
Vascular invasion: Yes vs No	**0.002**	2.6 (1.4‐4.8)	0.076	1.9 (0.94‐3.8)
TNM stage: III vs I‐II	**0.0004**	3.1 (1.7‐5.8)	**0.019**	2.2 (1.1‐4.4)
Histological grade: III vs I‐II	0.400	1.3 (0.69‐2.5)	NA	
PFKFB3: High vs Low	**0.0002**	3.5 (1.8‐6.6)	**0.006**	2.6 (1.3‐5.3)

Abbreviations: PFKFB3, phosphofructokinase‐2/fructose‐2,6‐bisphosphatase 3; HCC, hepatocellular carcinoma; TB, total bilirubin; ALT, alanine aminotransferase; PT, prothrombin time; HBV, hepatitis B virus; AFP, alpha‐fetoprotein; TNM, Tumor Node Metastasis.

**TABLE 3:** Univariate and multivariate COX regression analysis of clinicopathological features associated with disease‐free survival in HCC patients.

	Disease‐free survival			
	Univariate		Multivariate	
Clinicopathlogical Factors	*p* value	HR (95%CI)	*p* value	HR (95%CI)
Gender: female vs male	0.051	1.9 (1‐3.8)	NA	
Age(years): >50 vs ≤50	0.060	1.8 (0.98‐3.2)	NA	
HBV infection: Positive vs Negative	0.550	1.3 (0.59‐2.7)	NA	
TB(umol/L): >17.1 vs ≤17.1	0.700	0.85 (0.38‐1.9)	NA	
ALT(U/L): >40 vs ≤40	0.350	0.74 (0.39‐1.4)	NA	
PT(s): >14 vs ≤14	0.490	1.4 (0.57‐3.2)	NA	
AFP(ng/mL): >400 vs ≤400	0.400	1.3 (0.71‐2.3)	NA	
Tumor size(cm): >5 vs ≤5	**0.001**	2.8 (1.5‐5.2)	0.150	1.7 (0.82‐3.6)
Tumor number: Multiple vs Solitary	**0.043**	2.2 (1‐4.8)	0.840	1.1 (0.44‐2.7)
Vascular invasion: Yes vs No	**0.003**	2.4 (1.3‐4.4)	0.420	1.3 (0.66‐2.7)
TNM stage: III vs I‐II	**0.001**	2.8 (1.5‐5.3)	0.097	1.9 (0.89‐4)
Histological grade: III vs I‐II	0.1	1.7 (0.9‐3.3)	NA	
PFKFB3: High vs Low	**0.0007**	3 (1.6‐5.6)	**0.014**	2.3 (1.2‐4.4)

Abbreviations: PFKFB3, phosphofructokinase‐2/fructose‐2,6‐bisphosphatase 3; HCC, hepatocellular carcinoma; TB, total bilirubin; ALT, alanine aminotransferase; PT, prothrombin time; HBV, hepatitis B virus; AFP, alpha‐fetoprotein; TNM, Tumor Node Metastasis.

## 4 DISCUSSION

Although radiotherapy and cytotoxic chemotherapy have become the dominant methods in non‐surgical treatment of tumours, their clinical efficacy is still unsatisfactory due to chemoradiotherapy resistance.^39^, ^40^ Tumour microenvironment as a suitable fertile soil is not only conducive to tumour growth and progression, but also influences or modulates the sensitivity of tumour radiotherapy and cytotoxic chemotherapy.^41^ Hypoxia, immunosuppression and metabolic abnormalities have been substantiated to exert great impacts on chemoradiotherapy resistance.^41^, ^42^, ^43^, ^44^, ^45^, ^46^, ^47^ However, the effects of biomechanical stimuli especially matrix stiffness on chemoradiotherapy resistance and their underlying mechanism are largely undefined. Our previous studies have demonstrated that matrix stiffness augments the aggressiveness of HCC and facilitates the formation of lung pre‐metastatic niche.^28^, ^29^, ^48^ Moreover, matrix stiffness appears to weaken oxaliplatin‐induced apoptosis in HCC cells,^23^ suggesting a possible correlation between matrix stiffness and DNA damage repair in HCC. Besides its metabolic enzyme function, glycolytic enzymes can also perform non metabolic functions in tumour cells such as anti‐apoptosis, histone posttranslational modifications, transcription factors and cofactors, and protein kinase activity.^27^ This discovery prompts us to infer that glycolytic enzymes may have a dual regulatory effect on proliferation and survival of tumour cells. Accordingly, exploring the underlying mechanism of matrix stiffness‐potentiated HCC growth and radiotherapeutic resistance from the perspective of metabolic enzymes becomes the key goal of this study.

Since there is currently a lack of ideal matrix stiffness‐associated HCC animal models for observing the effect of matrix stiffness on radiation resistance, in vivo validation analysis of matrix stiffness‐mediated radiotherapeutic resistance has always been a technical challenge in experimental research. In this study, we creatively developed irradiated HCC cells/the unirradiated HCC cells‐derived HCC animal models with normal and high liver stiffness backgrounds. By observing the fold change of tumour growth, and combining analysis of Ki67 expression and TUNEL assay, we concluded that matrix stiffness obviously attenuated radiotherapeutic effect and enhanced cell survival from DNA damage. Similarly, analysis of cell assays also supported the conclusion that matrix stiffness promote cell survival from DNA damage. Other few studies also provide phenotypic evidence and indicate that higher matrix stiffness weakens the sensitivity of cytotoxic chemotherapy drugs.^49^, ^50^, ^51^ However, whether non‐enzyme function of metabolic enzyme contribute to matrix stiffness‐caused radiotherapy resistance has not been reported yet. In addition, the data in the study also suggested that matrix stiffness facilitated the growth of HCC tumours and promoted the proliferation of HCC cells, in agreement with our previous findings in buffalo rat HCC models with different liver stiffness backgrounds.^29^ Due to the prominent role of glycolytic enzymes in proliferation and survival of tumour cells,^24^, ^52^ glycolytic enzymes are likely to have the dual effects to mediate or participate in matrix stiffness‐potentiated proliferation and radiotherapeutic resistance.

By comparative analysing the expressions of key glycolytic enzymes in HCC cells cultured on different stiffness substrates, only PFKFB3 expression was found to be remarkably upregulated, while the expressions of other metabolic enzymes remained unchanged. Simultaneously, as matrix stiffness increased, glycolytic level of HCC cells and their proliferation were also obviously enhanced, and matrix stiffness‐promoted proliferation and glycolysis were effectively reversed by PFKFB3 knockdown. Thereby, matrix stiffness‐improved glycolytic level and proliferation in HCC cells was mainly attributed to PFKFB3 upregulation. In addition to promoting tumour proliferation, PFKFB3 upregulation also improves proliferation in benign tumours^53^ addressing the important role of PFKFB3 in cell proliferation. High‐stiffness substrate served as a potent inducer of PFKFB3, resulting in elevated protein levels, but failed to change its mRNA expression in HCC cells, implying that matrix stiffness‐caused PFKFB3 upregulation may be associated with its ubiquitination modification. We further disclosed the positive role of high stiffness stimulation in impeding PFKFB3 ubiquitination, and identified NEDD4 as an E3 ubiquitin ligase of PFKFB3. Moreover, we elucidated the regulatory mechanism by which high stiffness stimulation downregulated NEDD4 expression. Importantly, we confirmed the effects of NEDD4 knockdown on PFKFB3 ubiquitination and expression, proposing that NEDD4 mediates matrix stiffness‐caused PFKFB3 upregulation by influencing its ubiquitination level. Another study shows that UBC9‐induced‐PFKFB3 SUMOylation competitively prevents PFKFB3 polyubiquitination to promote aerobic glycolysis and proliferation of glioblastoma cells,^54^ also highlighting the role of protein posttranslational modification in modulating PFKFB3 expression and glycolysis.

Homologous recombination repair (HR) and non‐homologous end‐joining (NHEJ) are the two most common DNA repair pathways for DNA double‐strand breaks. It is reported that suppression of PFKFB3 or drug inhibition of PFKFB3 increases γ‐H2AX expression but decrease RAD51 expression in some cancer cell types.^55^, ^56^ Also, silencing PFKFB3 inhibits AKT phosphorylation and reduce ERCC1 expression in HCC cells, and thereby disrupts the function of DNA repair.^57^ Recently, a lietrature suggests that PFKFB3 can be rapidly recruited to the site of DNA damage induced by ionizing radiation, co‐localized with DNA damage marker and HR repair proteins, and thereby participating DNA repair.^58^ However, no reports are found about PFKFB3 nuclear translocation participating in matrix stiffness‐potentiated DNA repair through the NHEJ pathway. Here, we provided three experimental results including subcellular localization, tissue IHC and nuclear protein analysis to clarify that high matrix stiffness can prominently promote PFKFB3 nuclear translocation in the irradiated HCC cells. For the unirradiated HCC cells, total expression of PFKFB3 in high‐stiffness group was obviously higher than that in low‐stiffness group. Because less DNA damage occurs in HCC cells in the absence of radiation. PFKFB3 as a metabolic enzyme mainly participate in glycolysis. Most of metabolic enzyme PFKFB3 are mainly distributed at the cytoplasm, and only a small portion can be observed in the nucleus. Additionally, in high‐stiffness group without IR, PFKFB3 expression was upregulated, and their level in the nucleus was also increased slightly correspondingly. The nuclear PFKFB3 may be engaged in regulating other nuclear processes, for instance: nuclear PFKFB3 has been documented to regulate cell cycle progression.^59^ Following irradiation, high‐stiffness substrate synergized with DNA damage to induce a pronounced nuclear enrichment of PFKFB3, which was significantly greater than that observed in irradiated cells on low‐stiffness substrate. However, our data also indicate that this stiffness‐induced translocation, while significant, is quantitatively lower and functionally insufficient to enhance DNA repair in the absence of the specific activating signal, which is ionizing radiation. This explains why we observe no difference in DNA repair between stiffness conditions without IR.

Besides, importazole intervention effectively attenuated PFKFB3 nuclear translocation and DNA repair ability of the irradiated HCC cells on high‐stiffness substrate, also supporting that PFKFB3 nuclear translocation is crucial to matrix stiffness‐regulated DNA damage repair. Interaction site mutation analysis between Ku70 and PFKFB3 in the nucleus suggested that NHEJ pathway is responsible for matrix stiffness‐strengthened DNA repair.

PFKFB3 inhibitor has been reported to inhibit the progression of several types of cancer.^60^, ^61^ However, whether PFKFB3 inhibitor can be applied in clinical practice still faces a series of challenges. Considering that PFKFB3 plays a role in a normal glucose metabolism, intervention targeting PFKFB3 may influence and disrupt normal physiological function. Additionally, although PFKFB3 inhibitors have inhibition effects on tumour growth, they do not necessarily block PFKFB3 nuclear translocation and ameliorate radiotherapy resistance. Because there exist many target proteins for E3 ubiquitin ligase NEDD4, feasibility of NEDD4 intervention in anti‐cancer therapy is still needed further confirmation in the future.

Here, we mentioned several limitations inherent to this study. Despite we presented a wealth of evidence to clarify the contribution of matrix stiffness to PFKFB3 upregulation and its mechanism, in vivo functional validation of PFKFB3 in animal model is still lacking and further verification is needed. Secondly, in the irradiated HCC cells, PFKFB3 interacting with Ku70 mediated matrix stiffness‐enhanced DNA repair ability, however, the existence of other DNA repair pathways still cannot be ruled out.

**FIGURE 7:**
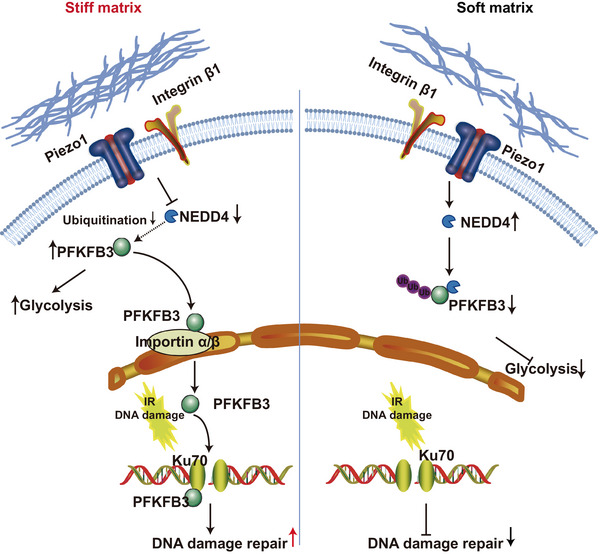
A diagram illustrating the underlying mechanism of matrix stiffness‐mediated glycolysis and DNA damage repair in HCC. Matrix stiffness suppressed PFKFB3 ubiquitination by downregulating E3 ubiquitin ligase NEDD4 expression, and then enhanced the stability of PFKFB3 protein to increases glycolysis. Simultaneously, matrix stiffness apparently strengthened the DNA damage repair ability of the irradiated HCC cells through PFKFB3 nuclear translocation and interacting with Ku70.

In conclusion, we have delineated a stiffness‐triggered pathway that co‐opts PFKFB3 expression and localization to fuel HCC growth and compromise radiotherapy efficacy (Figure 7). Specifically, matrix stiffness suppressed PFKFB3 ubiquitination by downregulating NEDD4 expression, and then enhanced the stability of PFKFB3 protein to increases glycolysis, ultimately promoted HCC growth and proliferation (Figure 7). Simultaneously, matrix stiffness apparently strengthened the DNA repair ability of the irradiated HCC cells through PFKFB3 nuclear translocation, and PFKFB3 interacting with Ku70 in the nucleus contributes to their DNA repair ability (Figure 7).

## AUTHOR CONTRIBUTIONS

All authors contributed to the study conception and design. Fan Wang, Taiwei Sun and Jiefeng Cui proposed conceptualizations and designed the study. Mimi Wang and Jiajun Li performed the experiments and data analysis. Jiali Qian, Xi Zhang, Miao Li, Yingying Zhao and Taiwei Sun helped finish the animal experiments. Miao Li and Rongxin Chen helped complete clinical samples collection and analysis. Zhiming Wang, Kun Guo, Dongmei Gao, Yan Zhao, Rongxin Chen and Zhenggang Ren provided guidance and suggestion for the experiments. Mimi Wang wrote the original draft, Jiefeng Cui revised it. All the authors read and approved the final manuscript.

## ETHICS APPROVAL

HCC tissues and clinical data used in the study were approved by the Ethics Committee of the Zhongshan Hospital of Fudan University (B2024‐359R). All the animal care and experimental procedures were approved by the Institutional Animal Care and Use Committee of Zhongshan Hospital of Fudan University (2020‐145).

## CONFLICT OF INTEREST STATEMENT

The authors declare no conflicts of interest.

## Supporting information



Supporting Information

## Data Availability

All data relevant to this study are available from the corresponding author on reasonable request.
