# Supplementary material for: Metabolic enzyme PFKFB3 mediates matrix stiffness‐potentiated tumour growth and radiotherapeutic resistance in HCC
Source: Clin Transl Med. 2025 Nov 25;15(12):e70509. doi: 10.1002/ctm2.70509 (PMC12647364; doi:10.1002/ctm2.70509)
Supplement: Supplementary file 1 — Supporting Information [file CTM2-15-e70509-s001.docx]

**Supplemental information**

**
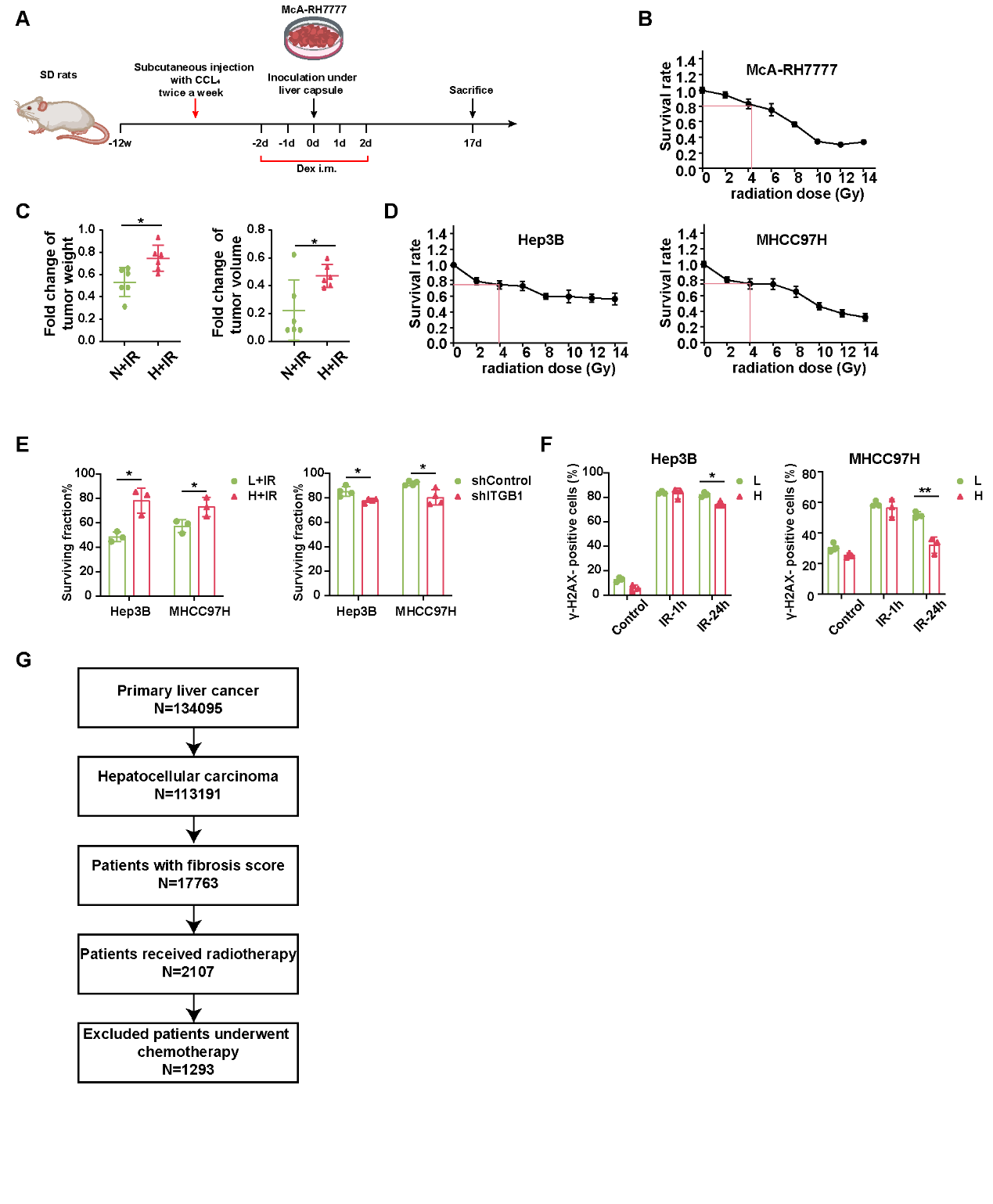
**

**Figure S1| Increased matrix stiffness potentiates tumor growth and radiotherapeutic resistance in HCC. (A)** Schematic diagram of HCC model construction in SD rats with high or normal liver stiffness background. CCL_4_**,** Carbon Tetrachloride; Dex, Dexamethasone; i.m., intramuscular injection. **(B)** The dose-survival curve of McA-RH7777 cells exposed to different irradiation doses (0Gy, 2Gy, 4Gy, 6Gy, 8Gy, 10Gy, 12Gy, and 14Gy). **(C)** The fold change of tumor weight and tumor volume from irradiated HCC cells in normal and high liver stiffness groups. **(D)** The dose-survival curve of Hep3B and MHCC97H cells exposed to different irradiation doses (0Gy, 2Gy, 4Gy, 6Gy, 8Gy, 10Gy, 12Gy, and 14Gy). **(E)** The survival fraction of HCC cells cultured on low-stiffness and high-stiffness substrates 7 days after exposure to irradiation (left panel). The survival fraction of HCC cells with integrin β1 knockdown cultured on high-stiffness substrates 7 days after exposure to irradiation (right panel). Cell viability was detected by CCK-8 assay. **(F)** Quantification of percentage of γ-H2AX positive (n>100 cells/treatment) HCC cells on low-stiffness and high-stiffness substrates 1 h and 24 h after irradiation, illustrated in Figure 1D. Cell with >10 γ-H2AX foci was taken as positive. **(G)** Flow chart depicting the selection of HCC patients from the SEER database for evaluating the effect of different fibrosis stages on radiotherapy resistance. L, low-stiffness substrate (6 kPa); H, high-stiffness substrate (16 kPa). IR, irradiation. shControl, empty vector; shITGB1, Integrin β1 knockdown. Values represent mean (SD); *p<0.05, **p<0.01, ***p<0.001, ****p < 0.0001; two-tailed Student’s t-test.

**
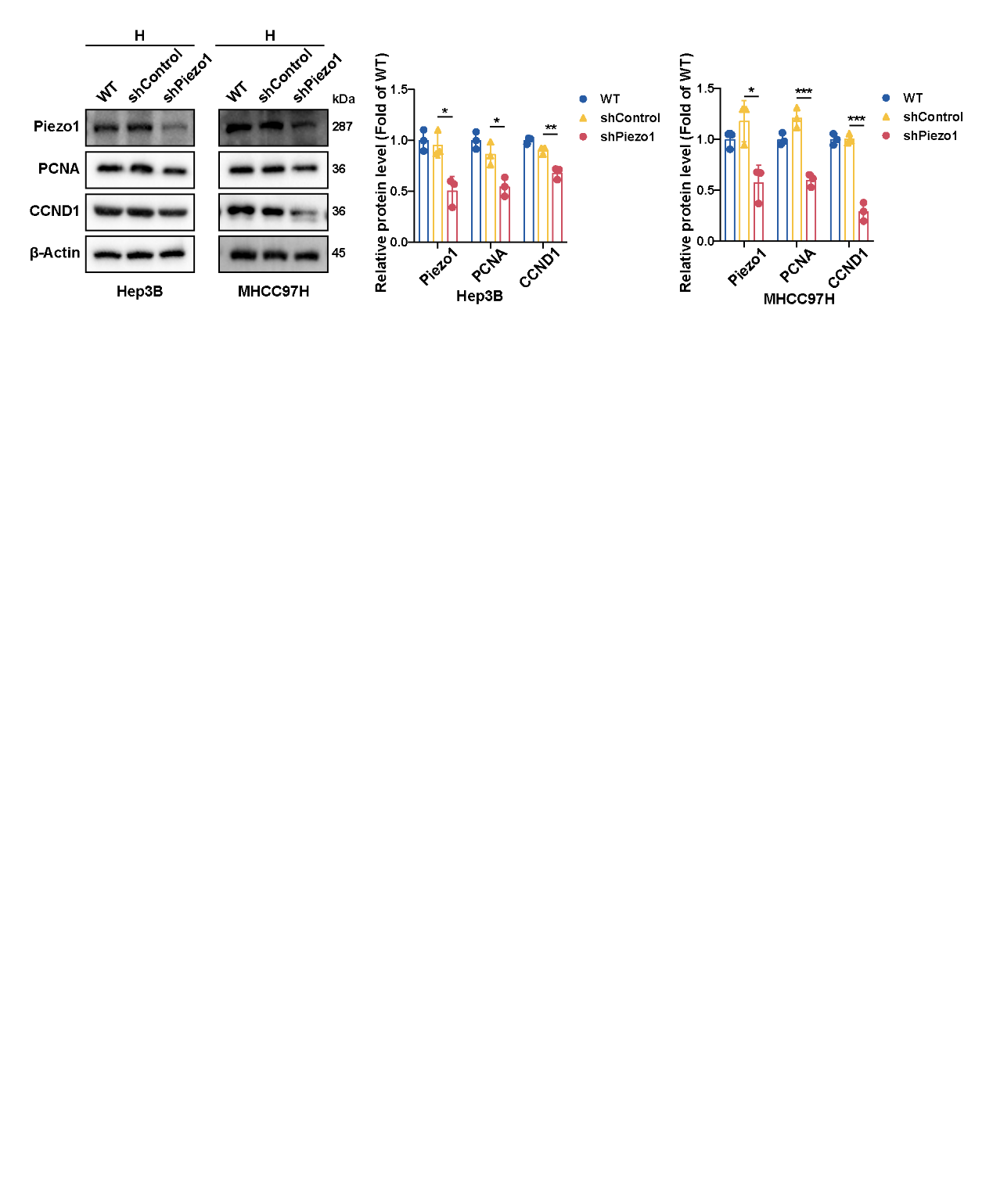
**

**Figure S2|** **Expressions of PCNA and CCND1 in HCC cells with shPiezo1 grown on high-stiffness substrates.** H, high-stiffness substrate (16 kPa). WT, wild type; shControl, empty vector; shPiezo1, Piezo1 knockdown. Values represent mean (SD); *p<0.05, **p < 0.01, ***p < 0.001, two-tailed Student’s t-test.

**
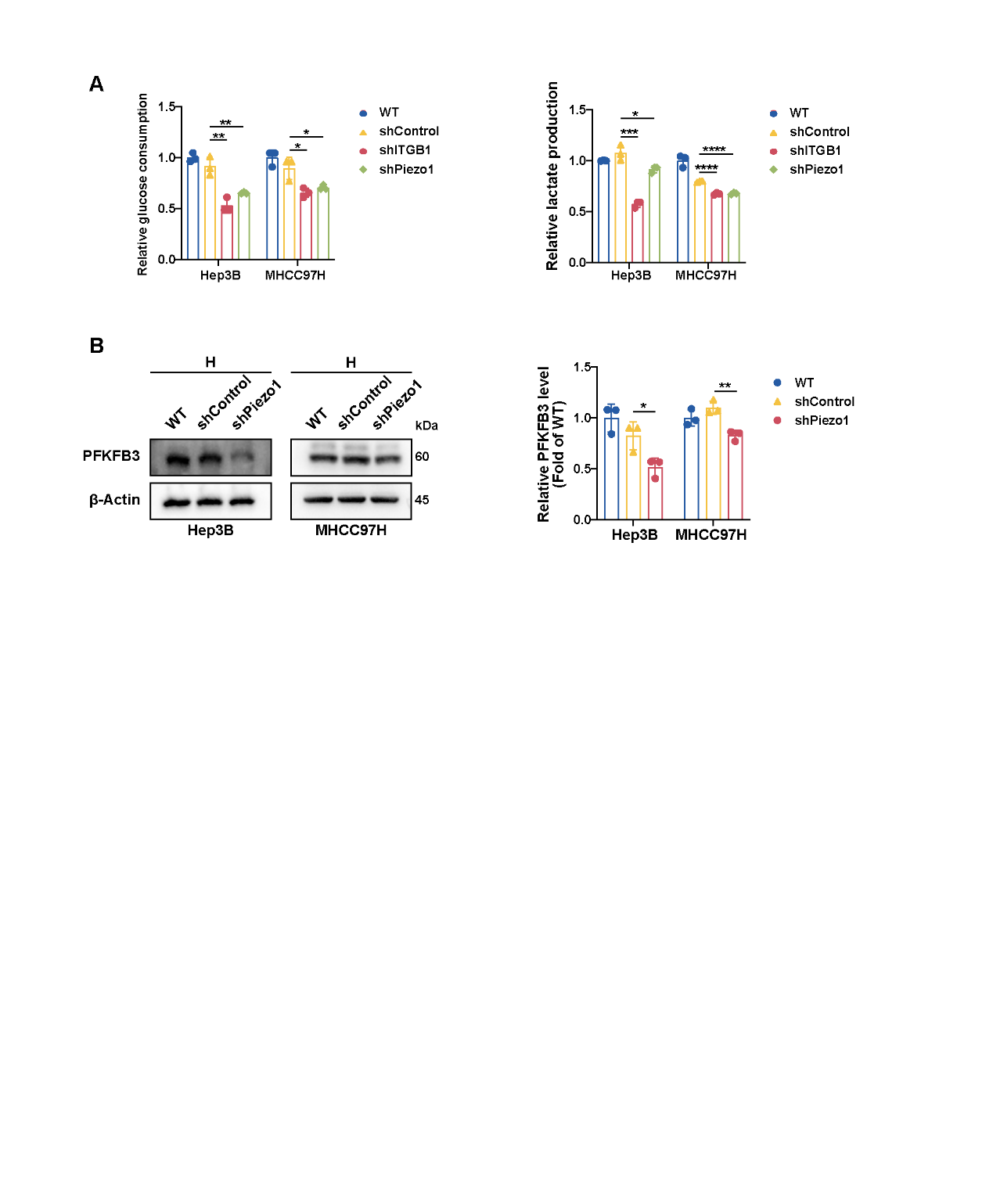
**

**Figure S3| Metabolic enzyme PFKFB3 participates in matrix stiffness-mediated effect on proliferation and radiotherapeutic resistance in HCC.** **(A)** Knockdown of integrin β1 or Piezo1 distinctly attenuated high stiffness stimulation-caused glucose consumption and lactate production. **(B)**

Expression of PFKFB3 in HCC cells with shPiezo1 grown on high-stiffness substrate. H, high-stiffness substrate (16 kPa). WT, wild type; shControl, empty vector; shITGB1, Integrin β1 knockdown; shPiezo1, Piezo1 knockdown. Values represent mean (SD); *p<0.05, **p < 0.01, ***p < 0.001, ****p < 0.0001; two-tailed Student’s t-test.

**
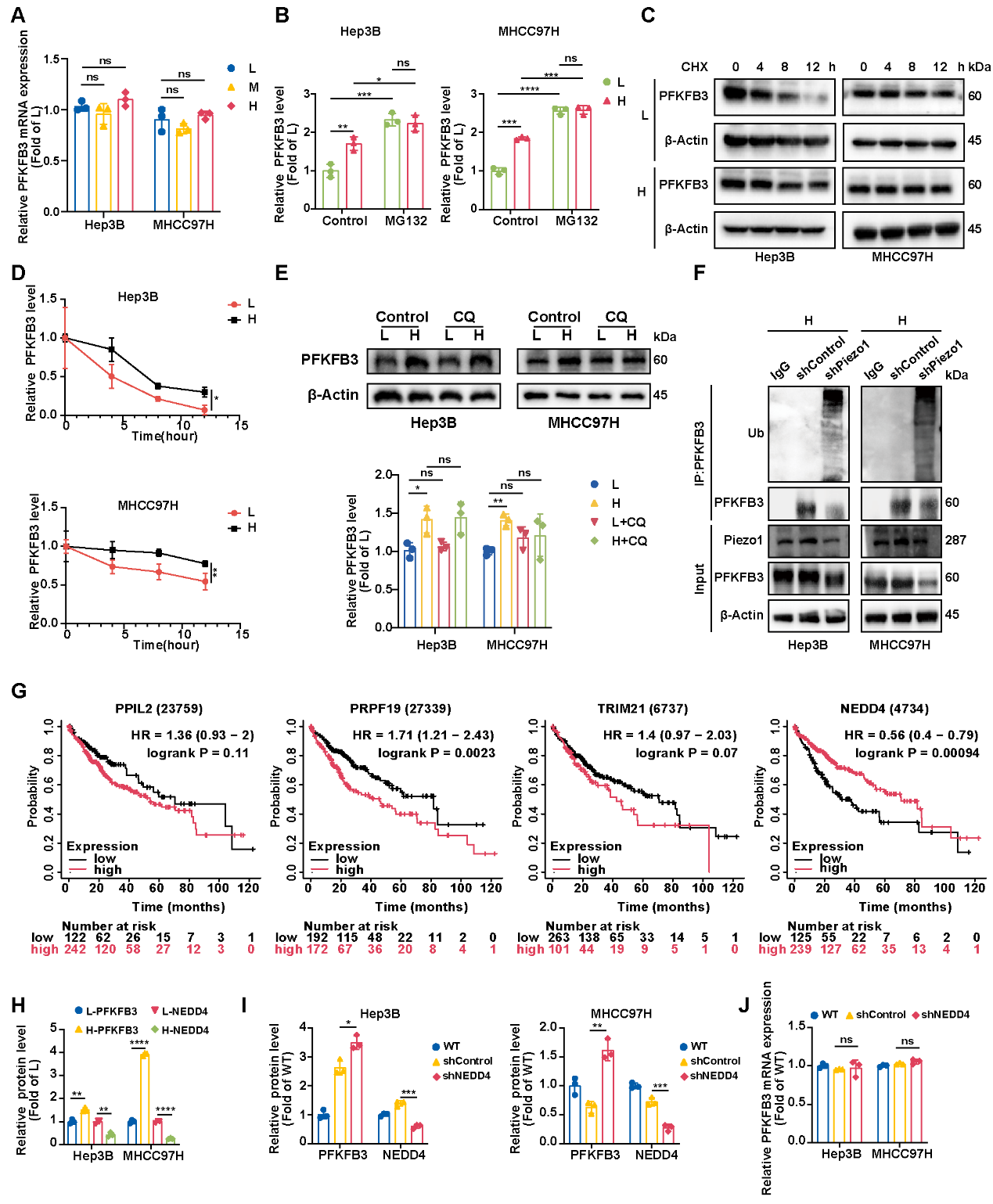
**

**Figure S4| High stiffness stimulation significantly improves PFKFB3 expression in HCC cells by lessening its ubiquitination level. (A)** The mRNA expression of PFKFB3 in HCC cells cultured on different stiffness substrates. **(B)** Quantification of PFKFB3 protein expression in HCC cells grown on low- and high-stiffness substrates under intervention of MG132 (10μM) for 6 h, as illustrated in Figure 4A. Control, DMSO. **(C)** The degradation of PFKFB3 in HCC cells on low- and high-stiffness substrates. Cells were intervened with CHX (100μg/mL) and collected at the indicated times. Control, DMSO; CHX, Cycloheximide. **(D)** Quantification of PFKFB3 expression, illustrated in Figure S4C. Two-way ANOVA, *p < 0.05,**p < 0.01.**(E)** Abundance of PFKFB3 expression of HCC cells on different stiffness substrates treated with CQ (10μM) for 6 h. Control, DMSO; CQ, Chloroquine. **(F)** Abundance of polyubiquitinated PFKFB3 upon PFKFB3 pull-down using whole cell protein of HCC cells with shPiezo1 grown on high-stiffness substrates. **(G)** The overall survival curves of PPIL2, PRPF19, TRIM21, and NEDD4 in TCGA-HCC patients, applying Kaplan-Meier method and log-rank test. PPIL2, peptidylprolyl isomerase like 2; PRPF19, pre-mRNA processing factor 19; TRIM21, E3 ubiquitin ligase tripartite motif (TRIM)-containing protein 21; NEDD4, neural precursor cell expressed, developmentally down-regulated 4. **(H)** Quantification of PFKFB3 and NEDD4 expressions in HCC cells grown on low- and high-stiffness substrates, as illustrated in Figure 4E. **(I)** Quantification of PFKFB3 expression in HCC cells with shNEDD4 grown on low-stiffness substrate, as illustrated in Figure 4F. **(J)** The mRNA expression of PFKFB3 in HCC cells with shNEDD4 cultured on low-stiffness substrate. L, low stiffness substrate (6 kPa); M, medium stiffness substrate (10 kPa); H, high stiffness substrate (16 kPa). WT, wild type; shControl, empty vector; shPiezo1, Piezo1 knockdown; shNEDD4, NEDD4 knockdown. Values represent mean (SD); **p<0.01, ***p<0.001, ****p < 0.0001, ns: not significant; two-tailed Student’s t-test.

**
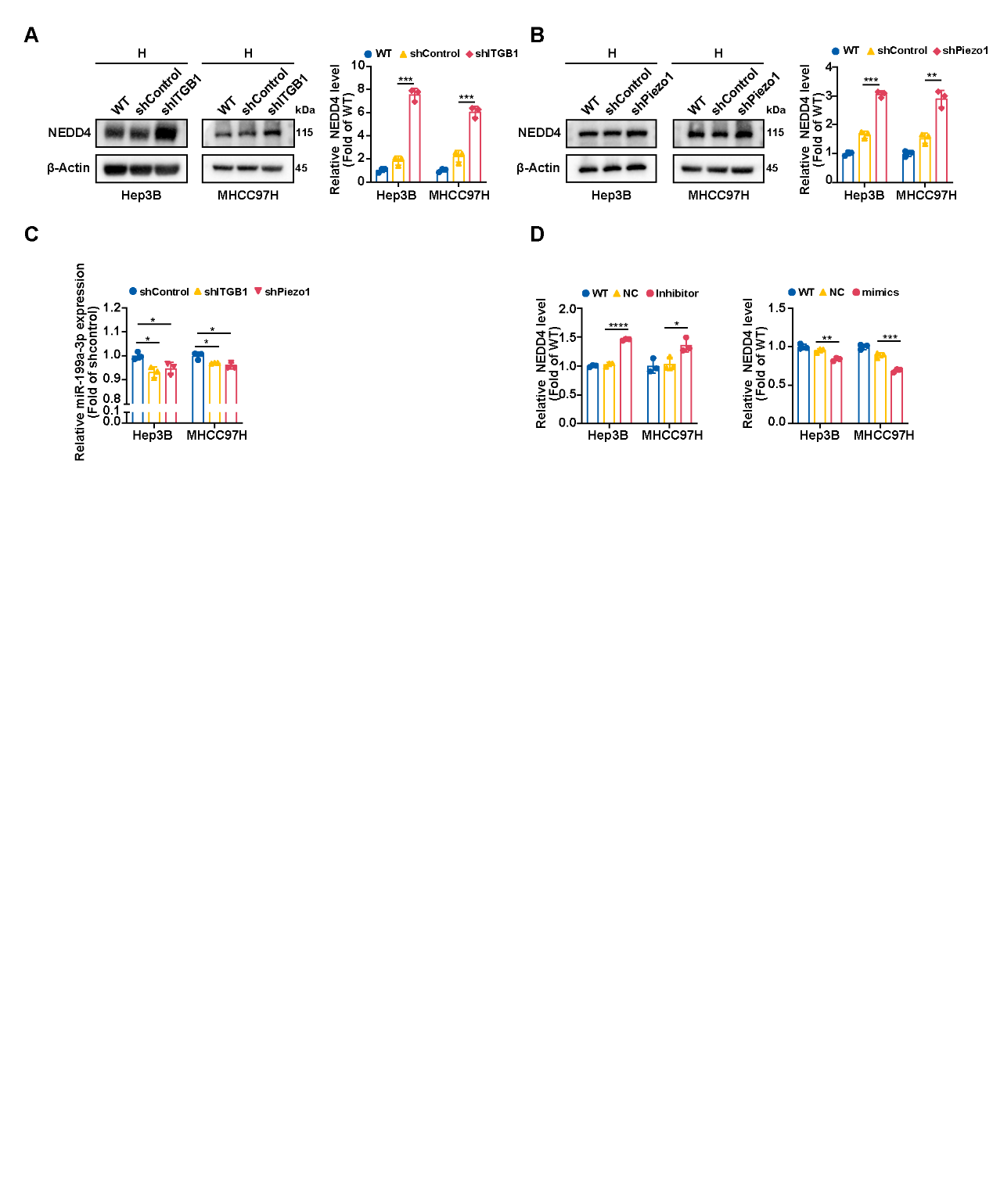
**

**Figure S5|High stiffness stimulation inhibits NEDD4 expression in HCC cells through** **miR-199a-3p. (A)** The expression of NEDD4 in HCC cells with shITGB1 grown on high-stiffness substrate. **(B)** The expression of NEDD4 in HCC cells with shPiezo1 grown on high-stiffness substrate. **(C)** Relative miR-199a-3p expression in HCC cells with shITGB1 or shPiezo1 on high-stiffness substrate. **(D)** Quantification of NEDD4 expression in HCC cells treated with miR-199a-3p inhibitor or mimics, as illustrated in Figure 4K. H, high stiffness substrate (16 kPa). WT, wild type; shControl, empty vector; shPiezo1, Piezo1 knockdown; shITGB1, Integrin β1 knockdown. Values represent mean (SD); *p<0.05, **p<0.01, ***p<0.001, ****p < 0.0001; two-tailed Student’s t-test.

**
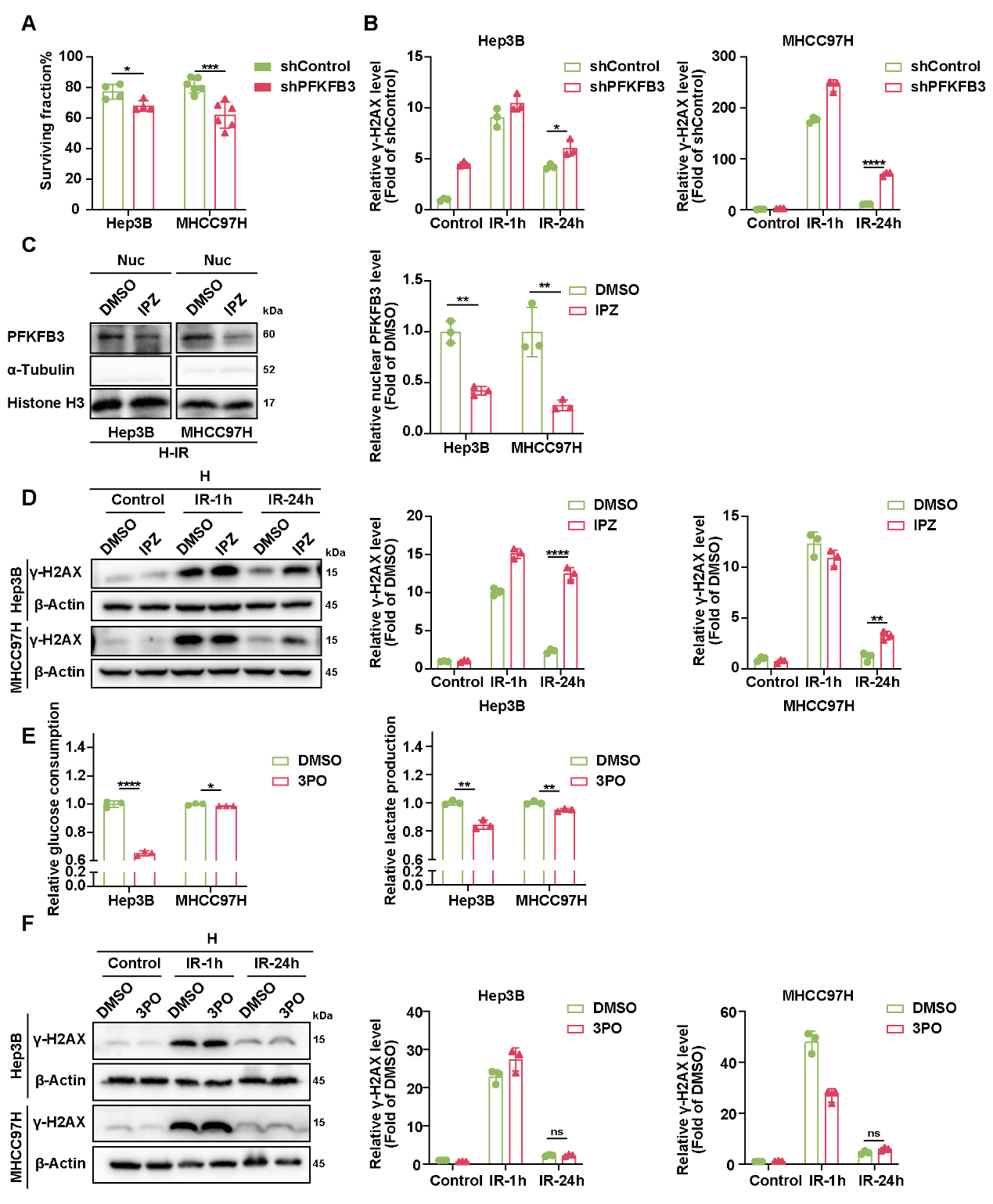
**

**Figure S6|Nuclear translocation of PFKFB3 contributed to matrix stiffness-induced DNA repair in HCC cells. (A)** The survival fraction of HCC cells with PFKFB3 knockdown cultured on high-stiffness substrate 7 days after irradiation. **(B)** Quantification of γ-H2AX level in HCC cells with PFKFB3 knockdown cultured on high-stiffness substrate 1 h and 24 h after irradiation, as illustrated in Figure 5C. **(C)** Importazole intervention (20μM) partially attenuated PFKFB3 nuclear translocation in the irradiated HCC cells on high-stiffness substrate. Nuc, nuclear; IPZ, importazole. **(D)** γ-H2AX expression in HCC cells with Importazole intervention grown on high-stiffness substrate 1 h and 24 h after irradiation. **(E)** Relative glucose consumption and lactate production in HCC cells on high-stiffness substrate, under exposure to 3PO (20uM) 6 h before irradiation. **(F)** γ-H2AX expression in HCC cells with 3PO intervention grown on high-stiffness substrate 1 h and 24 h after irradiation. shControl, empty vector; shPFKFB3, PFKFB3 knockdown. Values represent mean (SD); *p<0.05, **p<0.01, ***p<0.001, ****p<0.0001; ns, not significant; two-tailed Student’s t-test.

**
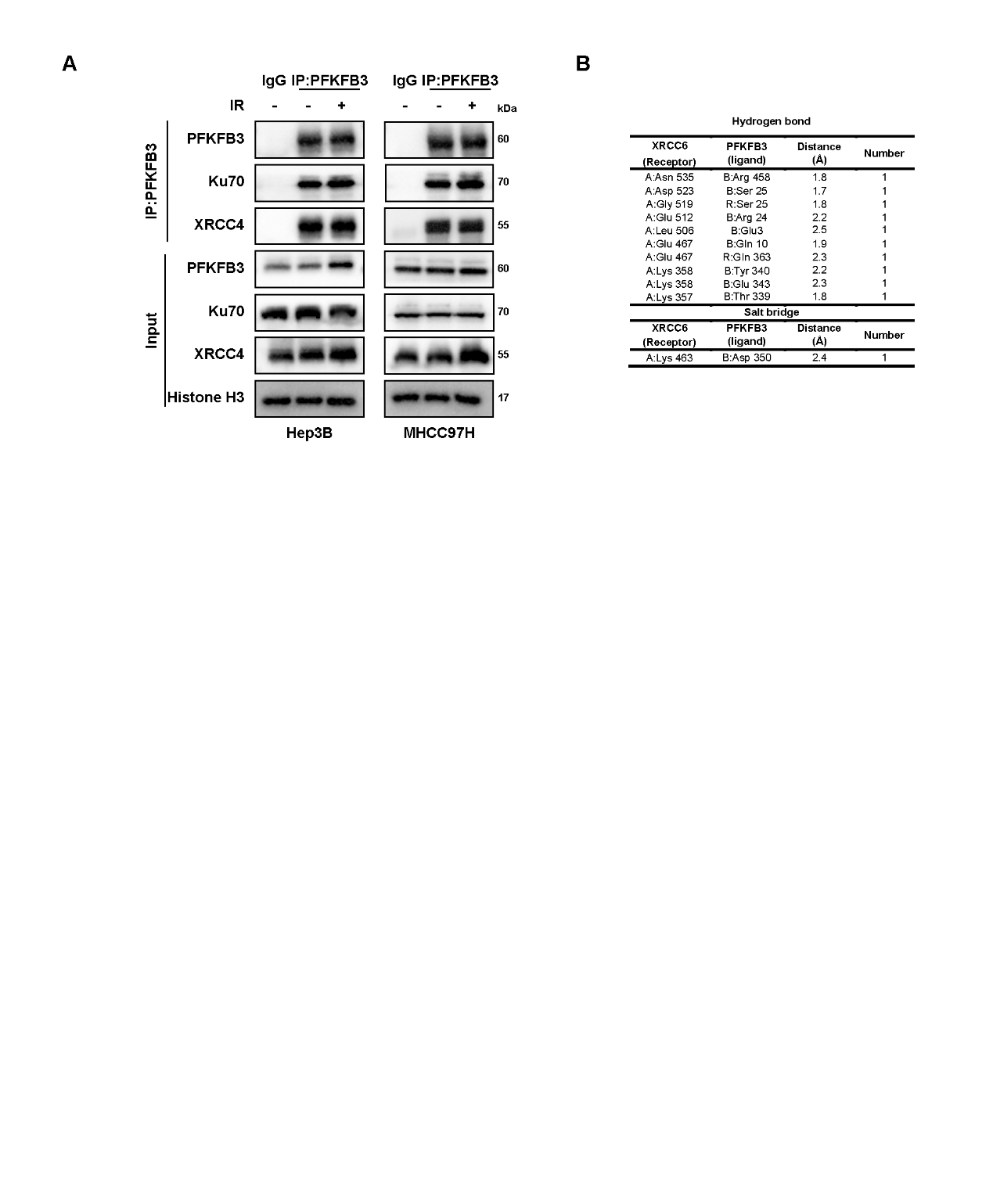
**

**Figure S7| PFKFB3 interacts with Ku70 to participate in DNA damage repair. (A)** Immunoblot of anti-PFKFB3 immunoprecipitation from nuclear protein of HCC cells 2 h after irradiation. **(B)** The non covalent interaction sites between PFKFB3 and XRCC6 analysed in Schrödinger software.


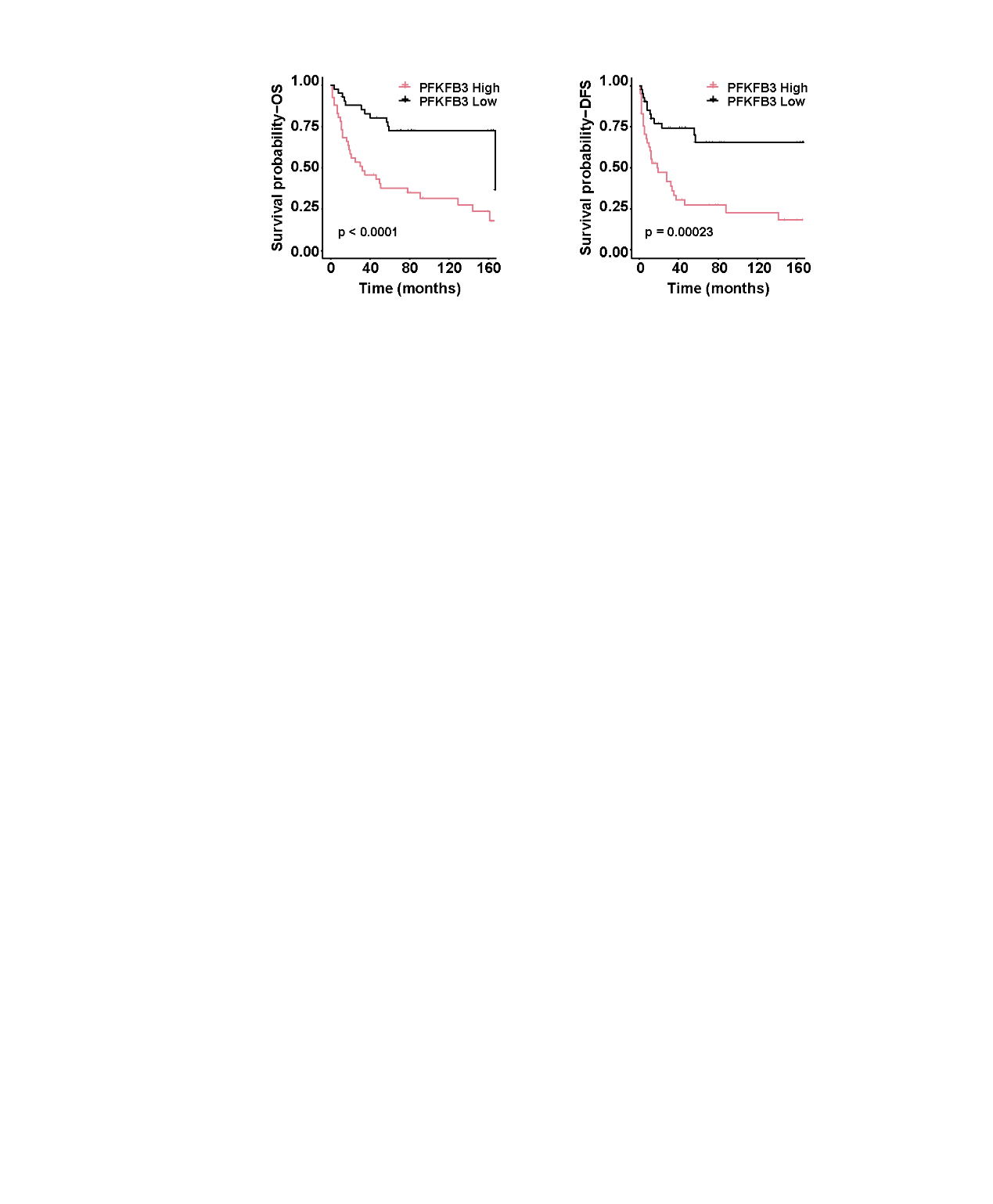


**Figure S8| Kaplan Meier curve comparing overall survival (OS, left panel) and disease-free survival (DFS, right panel) in PFKFB3^high^ and PFKFB3^low^ HCC patients.**

**Table S1. Univariate and multivariate analysis of OS in the radiotherapy population using the SEER database**

| **Clinicopathlogical Factors** | **Overall survival** | | | |  |
| --- | --- | --- | --- | --- | --- |
|  | **Univariate** | | **Multivariate** | |  |
|  | **p.value** | **HR (95%CI)** | **p.value** | **HR (95%CI)** | |
| Gender: male vs female | 0.096 | 1.17 (0.97-1.41) |  |  | |
| Age: ≤50 vs >50 | 0.261 | 0.78 (0.50-1.20) |  |  | |
| Race |  |  |  |  | |
| Black |  | Reference |  |  | |
| White | 0.903 | 1.02 (0.79-1.31) |  |  | |
| Other | 0.071 | 0.75 (0.55-1.02) |  |  | |
| Tumor number: solitary vs multiple | 0.009 | 1.28 (1.06-1.54) | 0.058 | 1.20 (0.99-1.44) | |
| TNM |  |  |  |  | |
| IV |  | Reference |  |  | |
| III | <0.001 | 0.41 (0.32-0.51) | 0.002 | 0.58 (0.41-0.82) | |
| II | <0.001 | 0.19 (0.15-0.25) | <0.001 | 0.41 (0.28-0.60) | |
| I | <0.001 | 0.17 (0.13-0.21) | <0.001 | 0.32 (0.23-0.46) | |
| Surgery: no vs yes | <0.001 | 2.37 (1.84-3.05) | <0.001 | 2.03 (1.57-2.62) | |
| Bone metastases: no vs yes | <0.001 | 0.18 (0.14-0.23) | <0.001 | 0.50 (0.34-0.74) | |
| Lung metastases: no vs yes | <0.001 | 0.12 (0.08-0.18) | <0.001 | 0.41 (0.25-0.65) | |
| Tumor size: ≤50 vs >50 | <0.001 | 0.46 (0.40-0.54) | <0.001 | 0.63 (0.53-0.76) | |
| **FibrosisScore: Ishak 0-4 vs Ishak 5-6** | **0.019** | **0.79 (0.64-0.96)** | **0.003** | **0.73 (0.60-0.90)** | |

OS, Overall survival; SEER, Survival analysis in Surveillance, Epidemiology, and End Results; TNM, Tumor Node Metastasis.

**Table S2. Univariate and multivariate analysis of CSS in the radiotherapy population using the SEER database**

| **Clinicopathlogical Factors** | **Cancer Specific survival** | | | |  |
| --- | --- | --- | --- | --- | --- |
|  | **Univariate** | | **Multivariate** | |  |
|  | **p.value** | **HR (95%CI)** | **p.value** | **HR (95%CI)** | |
| Gender: male vs female | 0.184 | 1.15 (0.94-1.41) |  |  | |
| Age: ≤50 vs >50 | 0.870 | 0.96 (0.62-1.49) |  |  | |
| Race |  |  |  |  | |
| Black |  |  |  |  | |
| White | 0.647 | 1.07 (0.80-1.42) |  |  | |
| Other | 0.152 | 0.78 (0.55-1.10) |  |  | |
| Tumor number: solitary vs multiple | <0.001 | 1.52 (1.22-1.88) | 0.002 | 1.41 (1.13-1.75) | |
| TNM |  |  |  |  | |
| IV |  |  |  |  | |
| III | <0.001 | 0.38 (0.30-0.49) | <0.001 | 0.53 (0.37-0.75) | |
| II | <0.001 | 0.18 (0.14-0.23) | <0.001 | 0.38 (0.26-0.57) | |
| I | <0.001 | 0.14 (0.11-0.18) | <0.001 | 0.27 (0.18-0.38) | |
| Surgery: no vs yes | <0.001 | 2.42 (1.82-3.21) | <0.001 | 2.04 (1.53-2.72) | |
| Bone metastases: no vs yes | <0.001 | 0.16 (0.12-0.22) | <0.001 | 0.51 (0.35-0.76) | |
| Lung metastases: no vs yes | <0.001 | 0.11 (0.07-0.18) | 0.001 | 0.44 (0.27-0.72) | |
| Tumor size: ≤50 vs >50 | <0.001 | 0.42 (0.35-0.49) | <0.001 | 0.59 (0.48-0.72) | |
| **FibrosisScore: Ishak 0-4 vs Ishak 5-6** | **0.017** | **0.76 (0.61-0.95)** | **0.002** | **0.70 (0.56-0.88)** | |

CSS, Cancer Specific survival; SEER, Survival analysis in Surveillance, Epidemiology, and End Results; TNM, Tumor Node Metastasis.

**Table S3. The enzyme E3 ligases binding to PFKFB3 in HCC cells identified by IP/MS**

| Accession | protein name | # Peptides IgG | # PSM IgG | # Peptides P3 |  | # PSM P3 | Peptides P3/IgG |
| --- | --- | --- | --- | --- | --- | --- | --- |
| Q13356 | PPIL2 | 25 | 28 | 20 |  | 25 | 0.8 |
| P19474 | TRIM21 | 15 | 15 | 15 |  | 16 | 1 |
| Q9UMS4 | PRPF19 | 3 | 3 | 7 |  | 7 | 2.3 |
| A0A024R5S9 | NEDD4 | 1 | 1 | 5 |  | 5 | 5 |
| C9J494 | MKRN2 |  |  | 1 |  | 1 |  |
| H3BT29 | PML | 1 | 1 | 1 |  | 1 | 1 |
| D3DTY9 | TRIM25 | 1 | 1 | 1 |  | 1 | 1 |
| A0A0G2JIW2 | TRIM26 |  |  | 1 |  | 1 |  |
| H0Y612 | TRIM33 |  |  | 1 |  | 1 |  |
| Q2Q1W2 | TRIM71 | 1 | 1 | 1 |  | 1 | 1 |
| H0YCU8 | ZFPL1 | 1 | 1 | 1 |  | 1 | 1 |
| G3FDP5 | RCHY1 | 1 | 1 |  |  |  | 0 |
| H7C2Y1 | TRIP12 | 1 | 1 |  |  |  | 0 |
| A0A0A0MSW0 | UBR4 | 1 | 1 |  |  |  | 0 |
| Q75MX9 | VPS41 | 1 | 1 |  |  |  | 0 |
| LC-MS/MS, Liquid chromatography-tandem mass spectrometry; PSM, peptide spectrum match; IgG, Isotype control antibody; P3, PFKFB3 antibody. | | | | | | | |

**Supplemental Materials and Methods**

**Immunoblotting**

Protein was extracted from cells using RIPA lysis buffer (Beyotime) containing 1×phosphatase inhibitors cocktail (Roche, Basel, Switzerland) and 1mM phenylmethanesulfonyl fluoride (PMSF) (Beyotime). Protein samples in 1× protein loading buffer (Beyotime) were loaded to conduct electrophoresis. After electrophoresis, the separated proteins were transferred onto a PVDF membrane (Merck Millipore). Subsequently, the PVDF membrane was blocked in 5% non-fat milk and then incubated in diluted primary antibodies overnight at 4℃. The following primary antibodies were used: PCNA, CCND1, CCND3, CDK2 , CDK4, PFKP, HK2, LDHA，ITGB1, Ubiquitin, γ-H2AX (Cell Signaling Technology, 1:1000); PKM2, NEDD4, β-Actin, Histone H3, α-Tubulin, Ku70, XRCC4 (Proteintech, 1:1000); PFKFB3 (Abcam, 1:1000); Piezo1 (Abcam, 1:200). After washed three times with 1×TBST, the membrane was continued to incubated with corresponding diluted secondary antibodies (Jackson ImmunoResearch,1:5000) at room temperature for 1 hour. Eventually, the protein bands on the membrane were visualized using freshly prepared chemiluminescence (ECL) working solution (Tanon, Shanghai, China) and observed by an ECL imaging system (Tanon).

**Immunofluorescence assay**

Cells were fixed with 4% paraformaldehyde (Beyotime) for 20 minutes at room temperature, and permeabilized with 0.5% Triton X-100 (Beyotime) for 15 minutes at room temperature. After washed with 1× PBS three times, they were blocked in 1× PBS containing 2% bovine serum albumin (BSA) and 0.1% Triton X-100 for 1 hour at room temperature. After incubation with primary antibodies at 4°C overnight, the cells were thoroughly washed (3 × 5 min) with 0.1% Triton X-100. The used primary antibodies were as follows: Ki67 (Cell Signaling Technology, 1:1000), γ-H2AX (Cell Signaling Technology, 1:1000), PFKFB3 (Abcam, 1:200). Afterwards, the cells were incubated with Alexa-Fluor-594/488 conjugated secondary antibodies (Yeason, 1:200) in dark for 2 hours at room temperature. Cells were further counterstained with 4′, 6-diamidino-2-phenylindole (DAPI, Yeasen) for 5 minutes at room temperature. Images of the stained cells were acquired using Olympus microscope (Tokyo, Japan), and Operetta CLS™ high content imaging analysis system (Danaher, Waltham, USA).

**Co-immunoprecipitation (Co-IP)**

Protein was separated and obtained freshly from the cells using Western and IP lysis buffer containing 1× phosphatase inhibitors and PMSF (Beyotime), and its concentration was quantified by the bicinchoninic acid assay (BCA, Beyotime). PureProteome™ Protein A/G Mix Magnetic Beads (Merck Millipore) were washed and resuspended with 500 μL binding/wash buffer (1× PBST, PH7.4, containing 0.1% TWEEN20, Sangon Biotech). Then, primary antibodies (PFKFB3, Abcam; Ku70, Proteintech/Abclonal) were coupled to beads by continuous rotation mixing at 4 °C for 4 hours. Next, protein samples were added to the antibody-bound beads, and continuously mixed by rotation at 4 °C overnight. After wash, protein from beads were eluted and then solubilized in 1× SDS-PAGE loading buffer (Beyotime) for subsequent immunoblotting assay or mass spectrometry.

**Liquid chromatography-tandem mass spectrometry (LC-MS/MS)**

Protein samples acquired by the IP method described above were separated using polyacrylamide gel electrophoresis, and then the gel was stained with Coomassie blue staining solution (Beyotime). Protein bands in the gel were collected and enzymatically hydrolyzed into peptides using trypsin, which were extracted with 60% ACN/0.1% TFA and dried completely with a vacuum centrifuge. The obtained peptides were further detected using an MS spectrometer (nanoLC-QE, Thermo Fisher Scientific, Waltham, MA, USA). Raw data files of mass spectrometry were analyzed using Proteome Discoverer 1.4 software (Thermo Fisher), and peptide identification was performed using Sequest HT searching the human protein database- UniProt.

**Immunohistochemistry (IHC)**

Tissue slides were deparaffinized with xylol and dehydrated with ethanol. After antigen retrieval in boiled sodium citrate repair solution (Beyotime) and antigen blocking in blocking solution with 3% hydrogen peroxide and 3% BSA, the slides were incubated with primary antibodies (Ki67, Cell Signaling Technology, 1:1000; PFKFB3, Proteintech, 1:500; NEDD4, Proteintech, 1:500) for overnight at 4℃, and then treated with horseradish peroxidase (HRP)-conjugated antibodies. Tissue slide visualized by 3,3N-diaminobenzidine tertrahydrochloride (DAB) (Gene Tech), and hematoxylin counterstaining. TUNEL assays were conducted using DAB (SA-HRP) Tunel Cell Apoptosis Detection Kit (Servicebio, China) according to the manufacturer’s instruction. The stained slides were observed and photographed using the standard microscope (Olympus, Tokyo, Japan). The images obtained were analyzed by Image-Pro Plus 6.0 software.

**Cell counting kit-8 (CCK-8) assay**

A work solution with CCK-8 reagent (Beyotime) and complete culture medium was first prepared in a ratio of 10:100. Following cell seeding in a 96-well plate, 110 μL of the working solution was added to each well, followed by a 1-hour incubation at 37°C. The absorbance of each culture well was measured using 450nm wavelength on a microplate reader (Thermo Fisher Scientific).

**Real-time quantitative Polymerase Chain Reaction (qRT-PCR)**

Total RNA was extracted from the cells using Trizol (Sigma). For mRNA, reversed into complementary DNA (cDNA) using RevertAid Reverse Transcription kit (Thermo Fisher Scientific). Next, the cDNA mixed with SYBR Green Master Mix kit (Yeason). For miRNA , cDNA synthesis and PCR amplification were performed using the All-in-One™ miRNA qRT-PCR Assay Kit 2.0 (GeneCopoeia) following the manufacturer’s protocol.

Specific primers were used to amplify target sequences, and quantification was carried out on a real-time PCR instrument (Eppendorf Corporation, Hamburg, Germany). β-actin and U6 were used as internal reference genes for normalization of mRNA and miRNA expression levels, respectively. Relative gene expression was calculated using the 2^-ΔΔCt^ method. The primer sequences of target genes are as follows：Human PFKFB3 forward: CTCGCATCAACAGCTTTGAGG, reverse: TCAGTGTTTCCTGGAGGAGTC. Human β-Actin forward: AGGCCAACCGCGAGAAGATGACC, reverse: GAAGTCCAGGGCGACGTAGCAC. hsa-miR-199a-3p forward: ACAGUAGUCUGCACAUUGGUUA. Universal reverse primer From All-in OneTM miRNA qRT-PCR Detection Kit 2.0. Human U6 forward: GGAACGATACAGAGAAGATTAGC, reverse: TGGAACGCTTCACGAATTTGCG.

**Kaplan-Meier survival analysis**

The prognosis of candidate E3 ligases in HCC patients were analyzed online using the Kaplan–Meier Plotter (<http://kmplot.com/>), and the statistical analysis was carried out by log-rank test.

**Screening of candidate proteins binding to PFKFB3 in nuclear protein**

To screen the candidate proteins binding to PFKFB3 in the nucleus for DNA damage repair, the irradiated HCC cells (4Gy) were collected and their nuclear protein was extracted. The obtained nuclear protein was immunoprecipitated by PFKFB3 antibody (Abcam), and the captured proteins were analyzed by LC-MS/MS. Using peptide spectrum matches (PSMs) greater than 4 as the threshold, a total of 1005 PFKFB3-binding proteins in nuclear protein were selected. The molecular sets of the homologous recombinant (HR) and non-homologous end joining (NHEJ) pathway were downloaded from the Kyoto Encyclopedia of Genes and Genomes (KEGG, <https://www.genome.jp/kegg/>). Intersected with these sets of proteins participating in DNA damage repair, 4 nuclear PFKFB3-binding proteins were obtained.

**Survival analysis in Surveillance, Epidemiology, and End Results (SEER) database**

Prospectively collected data from SEER database maintained by the National Cancer Institute were used for analysis in this study. The database used in the study was the SEER 17 Regs Custom data (with additional treatment fields), Nov2022Sub (2000–2020). The inclusion criteria were as follows: (1) primary liver cancer, site code: C22.0; (2) hepatocellular carcinoma, histological codes:8170/3-8175/3 [in the light of the International Classification of Tumor Diseases Third Edition (ICD-O-3)]. (3) patients with complete fibrosis score. (4) patients received radiotherapy. The exclusion criteria were patients received chemotherapy. Kaplan Meier survival curves and compared using the log-rank test. Data analysis was performed using R Foundation Statistical software (R 3.2.1)
